# Fine mapping and identification of serum urate loci in American Indians: The Strong Heart Family Study

**DOI:** 10.1038/s41598-019-52924-w

**Published:** 2019-11-29

**Authors:** Geetha Chittoor, Karin Haack, Poojitha Balakrishnan, Christopher Bizon, Sandra Laston, Lyle G. Best, Jean W. MacCluer, Kari E. North, Jason G. Umans, Nora Franceschini, Gauri Prasad, Luis Macias-Kauffer, Teresa Villarreal-Molina, Dwaipayan Bharadwaj, Samuel Canizales-Quinteros, Ana Navas-Acien, Shelley A. Cole, V. S. Voruganti

**Affiliations:** 1 Department of Nutrition, and UNC Nutrition Research Institute, University of North Carolina at Chapel Hill, Kannapolis, NC USA; 2 Biomedical and Translational Informatics, Geisinger, Danville, PA USA; 3 Population Health Program, Texas Biomedical Research Institute, San Antonio, TX USA; 4 Department of Environmental Health Sciences, Columbia University Mailman School of Public Health, New York, New York, USA; 5 Renaissance Computing Institute, University of North Carolina, Chapel Hill, NC USA; 6 South Texas Diabetes and Obesity Institute, School of Medicine, University of Texas Rio Grande Valley, Brownsville, TX USA; 7 Missouri Breaks Industries Research Inc., Eagle Butte, SD USA; 8 Department of Epidemiology, University of North Carolina at Chapel Hill, Chapel Hill, NC USA; 9 Medstar Health Research Institute, Hyattsville, MD USA; 10 Genomics and Molecular Medicine Unit, CSIR-Institute of Genomics and Integrative Biology, New Delhi, 110 020 India; 11 Academy of Scientific and Innovative Research, CSIR-Institute of Genomics and Integrative Biology Campus, New Delhi, 110 020 India; 12 Laboratorio de Enfermedades Cardiovasculares, INMEGEN, Mexico City, 14610 Mexico; 13 Unidad de Genomica de Poblaciones Aplicada a la Salud Facultad de Quimica, UNAM-Instituto Nacional de Medicina Genomica, Mexico City, Mexico; 14 Systems Genomics Laboratory, School of Biotechnology, Jawaharlal Nehru University, New Delhi, 110 067 India

**Keywords:** Genome-wide association studies, Quantitative trait loci

## Abstract

While studies have reported genetic loci affecting serum urate (SU) concentrations, few studies have been conducted in minority populations. Our objective for this study was to identify genetic loci regulating SU in a multigenerational family-based cohort of American Indians, the Strong Heart Family Study (SHFS). We genotyped 162,718 single nucleotide polymorphisms (SNPs) in 2000 SHFS participants using an Illumina MetaboChip array. A genome-wide association analysis of SU was conducted using measured genotype analysis approach accounting for kinships in SOLAR, and meta-analysis in METAL. Our results showed strong association of SU with rs4481233, rs9998811, rs7696092 and rs13145758 (minor allele frequency (MAF) = 25–44%; *P* < 3 × 10^−14^) of solute carrier family 2, member 9 (*SLC2A9*) and rs41481455, rs2231142 and rs1481012 (MAF = 29%; p < 3 × 10^−9^) of ATP-binding cassette protein, subfamily G, member 2 (*ABCG2*). Carriers of G alleles of rs9998811, rs4148155 and rs1481012 and A alleles of rs4481233, rs7696092 and rs13145758 and rs2231142 had lower SU concentrations as compared to non-carriers. Genetic analysis of SU conditional on significant *SLC2A9* and *ABCG2* SNPs revealed new loci, nucleobindin 1 (*NUCB1*) and neuronal PAS domain protein 4 (*NPAS4*) (p <6× 10^−6^). To identify American Indian-specific SNPs, we conducted targeted sequencing of key regions of *SLC2A9*. A total of 233 SNPs were identified of which 89 were strongly associated with SU (p < 7.1 × 10^−10^) and 117 were American Indian specific. Analysis of key SNPs in cohorts of Mexican-mestizos, European, Indian and East Asian ancestries showed replication of common SNPs, including our lead SNPs. Our results demonstrate the association of SU with uric acid transporters in a minority population of American Indians and potential novel associations of SU with neuronal-related genes which warrant further investigation.

## Introduction

Hyperuricemia or elevated concentration of urate in serum (SU) is a risk factor for gout, hypertension, chronic kidney disease (CKD) and cardiovascular disease (CVD)^1–4^. Uric acid is the final product of purine metabolism in humans, and urate homeostasis involves balancing its production with secretion and reabsorption in the proximal convoluted tubule of kidneys^3,4^. The variation in SU concentration is under significant genetic influence and its pattern of inheritance suggests that many genes may influence it^1^. Correspondingly, the renal transport of urate involves several genes including solute carrier family 2, member 9 (*SLC2A9*), ATP-binding cassette ABC, subfamily G, member 2 (*ABCG2*), solute carrier family 22, members 11 and 12 (*SLC22A11* and *SLC22A12)*, solute carrier family 17, members 1, 3 and 4 (*SLC17A1*, *SLC17A3* and *SLC17A4)*, and solute carrier family 16, member 9 (*SLC16A9)*. Most of these genes have been associated with hyperuricemia^1–6^.

Both hyper and hypouricemia have been linked to increased risk for metabolic diseases. While hypouricemia has been linked to neurological disorders such as multiple sclerosis and Parkinson’s disease^7,8^, hyperuricemia is causal for gout and nephrolithiasis and seems to increase the risk for CKD and CVD^1–4^. Originally thought to be just a marker, SU’s role in development and progression of these diseases is being increasingly recognized^3^. While a recent review by Li *et al*.^9^ found no clear role for uric acid in metabolic diseases other than gout and nephrolithiasis, many studies including ours, have shown that gout patients and asymptomatic hyperuricemic individuals tend to be at high risk for CVD and CKD (Table 1)^10,11^. Therefore, it is essential to understand the genetic and environmental factors that affect the variation in SU. Even though genome-wide association studies (GWAS) have identified many SU-related loci, the majority of these studies have been conducted in European, African American and Asian populations^12–22^. To better understand genetic variation, biological significance and translation to human health, it is important to study ethnically diverse populations^23^. Further, the linkage disequilibrium (LD) pattern differences in ethnically diverse populations may offer a unique perspective on fine mapping of genetic loci.

**Table 1:** Hyperuricemia and cardiovascular disease risk factors in SHFS.

Phenotype*	Normouricemia	Hyperuricemia**	P value
**Anthropometric**			
Body weight (kg)	88.08 (24.1)	97.79 (23.8)	<0.0001
Waist circumference (cm)	103.57 (18.8)	107.59 (18.0)	<0.0001
BMI (kg/m^2^)	33.04 (8.2)	34.38 (7.3)	NS
**Lipids**			
Total cholesterol (mg/dl)	177.46 (34.6)	189.74 (42.3)	<0.0001
HDL cholesterol (mg/dl)	51.56 (14.6)	48.26 (14.1)	<0.0001
LDL cholesterol (mg/dl)	95.92 (28.1)	104.39 (31.8)	<0.0001
Triglycerides (mg/dl)	156.65 (129)	200.44 (254)	<0.0001
**ECHO measures**			
Heart rate (BPM)	66.99 (10.6)	67.38 (11.5)	NS
Left ventricular mass (g)	152.72 (39.0)	175.27 (41.7)	<0.0001
Relative wall thickness	0.298 (0.04)	0.301 (0.04)	0.030
Stroke volume (ml)	80.81 (14.5)	84.90 (15.1)	<0.0001
Ejection fraction	60.41 (5.7)	58.63 (5.8)	<0.0001
Cardiac output (ml/min)	5376.44 (1153)	5673,0.78 (1252)	<0.0001
Total peripheral resistance (dyne/cm/sec)	1410.02 (304)	1392.27 (316)	NS
**Blood pressure**			
Systolic (mmHg)	121.25 (17.2)	126.56 (15.8)	<0.0001
Diastolic (mmHg)	75.47 (11.0)	78.46 (11.6)	<0.0001
**Carotid measures**			
Left common carotid diastolic diameter (mm)	5.67 (0.7)	6.00 (0.7)	<0.0001
Right common carotid diastolic diameter (mm)	5.78 (0.7)	6.10 (0.7)	<0.0001
Left common carotid systolic diameter (mm)	6.40 (0.7)	6.78 (0.7)	<0.0001
Right common carotid systolic diameter (mm)	6.51 (0.7)	6.87 (0.8)	<0.0001
Left common carotid artery intimal medial thickness (mm)	0.66 (0.2)	0.69 (0.2)	<0.0001
Right common carotid artery intimal medial thickness (mm)	0.66 (0.2)	0.68 (0.2)	0.003

^*^Phenotypes are shown as means (SD); NS – Not significant.

**Hyperuricemia was defined as serum urate concentrations > 6 mg/dl in women and > 7 mg/dl in men.

American Indians are such a population that is understudied and underrepresented in genetic databases. The prevalence of CVD and CKD is high in American Indians with heart disease being the leading cause of death [https://www.cdc.gov/dhdsp/data_statistics/fact_sheets/fs_aian.htm]. The Strong Heart Family Study (SHFS) is a multigenerational family-based study of CVD in American Indians. This cohort has high rates of obesity, diabetes, CKD and CVD^24–26^. In addition, about 25% of individuals have hyperuricemia (SU > 6 mg/dl)^27^. Thus, our aim in this study was to identify the genetic loci that regulate SU concentrations in American Indians. The GWAS was first conducted in each of the three centers of the SHFS (Arizona, Oklahoma and Dakotas (North and South)), followed by a meta-analysis of all three centers. As a secondary aim, we aimed to identify American Indian-specific SNPs in *SLC2A9*, the gene most strongly associated with SU in this study.

## Results

The current study included 3000 SHFS participants (1282 men and 1718 women) from three study centers, Arizona, North and South Dakota (Dakotas) and Oklahoma. The mean SU concentrations were 5.14 ± 1.5 mg/dl (4.6 ± 1.3 mg/dl in women and 6.0 ± 1.4 mg/dl in men); 4.9 ± 1.5 mg/dl, 5.2 ± 1.5 mg/dl, 5.3 ± 1.5 mg/dl in Arizona, Dakotas and Oklahoma respectively. Genetic analysis was conducted using rank-inverse-normal transformed SU concentrations, which were regressed on covariates such as age, sex, and their interactions, diabetes status, and medications^27,28^.

### MetaboChip data analysis

Metabochip genotyping was conducted in a subset of 2000 SHFS (Arizona = 300, Dakotas = 850, Oklahoma = 850) participants who were free of diabetes at visit 1. The final data set included 162,718 autosomal SNPs. MetaboChip data analysis, conducted in each of the three SHFS centers, revealed significant associations of SU with *SLC2A9* SNPs (*P* < 4 × 10^−7^); rs13145758, rs9998811, rs7862063 in Arizona and rs4481233 in Dakotas and Oklahoma. The minor allele frequencies (MAFs) ranged between 25 and 44%, and the effect sizes (proportion of the residual phenotypic variance that is explained by the minor allele of the SNP) ranged between 4 and 6% (Table 2). The most significant SNP in Arizona was rs13145758 whereas it was rs4481233 in Oklahoma and the Dakotas. Several other SNPs showed associations at *P* < 1 × 10^−5^ including rs4148155, rs1481012 and rs2231142 of *ABCG2*.

**Table 2:** Genome-wide association analysis of serum urate stratified by center.

SNP	Minor Allele	MAF	*P*-value	β	SE	Effect Size	Gene	Gene loc	Chr	Chr pos hg18
**Arizona**										
rs13145758	A	0.43	1.1 × 10^−10^	−0.35	0.05	0.06	*SLC2A9*	Intron	4	9981997
rs9998811	G	0.44	7.4 × 10^−10^	−0.33	0.05	0.06	*SLC2A9*	Intron	4	9966477
rs7862063	G	0.41	4. 5 × 10^−8^	0.29	0.05	0.04	—	—	9	110002036
rs4481233	A	0.41	2.3 × 10^−6^	−0.26	0.05	0.03	*SLC2A9*	Intron	4	9956079
rs6688009	A	0.01	9. 1 × 10^−6^	1.07	0.24	0.03	*PPAP2B*	Intron	1	24593576
**Dakotas**										
rs4481233	A	0.25	6. 7 × 10^−8^	−0.29	0.05	0.04	*SLC2A9*	Intron	4	9956079
rs7696092	C	0.19	1. 6 × 10^−6^	−0.29	0.06	0.03	*SLC2A9*	Intron	4	10025320
rs179409	G	0.45	3.4 × 10^−6^	−0.23	0.05	0.03	*KCNQ1*	Intron	11	2483882
rs9998811	A	0.41	4.2 × 10^−6^	−0.22	0.05	0.03	*SLC2A9*	Intron	4	9966477
rs13145758	G	0.40	5.1 × 10^−6^	−0.22	0.05	0.03	*SLC2A9*	Intron	4	9981997
rs7947391	A	0.22	5.6 × 10^−6^	−0.26	0.06	0.02	*NPAS4*	Intron	11	66186882
**Oklahoma**										
rs4481233	A	0.30	3.3 × 10^−9^	−0.30	0.05	0.06	*SLC2A9*	Intron	4	9956079
rs9998811	A	0.46	5.8 × 10^−7^	−0.23	0.05	0.04	*SLC2A9*	Intron	4	9966477
rs4148155	G	0.20	1.1 × 10^−7^	0.29	0.06	0.03	*ABCG2*	Intron	4	89054667
rs2231142	A	0.20	1.1 × 10^−6^	0.29	0.06	0.03	*ABCG2*	Missense	4	89052323
rs1481012	G	0.20	1.1 × 10^−6^	0.29	0.06	0.03	*ABCG2*	Intron	4	89039082
rs7696092	C	0.29	2.1 × 10^−6^	−0.25	0.05	0.04	*SLC2A9*	Intron	4	10025320
rs13145758	G	0.45	2.7 × 10^−6^	−0.22	0.05	0.03	*SLC2A9*	Intron	4	9981997
rs746075	A	0.36	2.8 × 10^−6^	0.22	0.05	0.04	*NUCB1*	Intron	19	49416936

^*^SNP: single nucleotide polymorphism; MAF: minor allele frequency; P-value: P-values from measured genotype analysis; β: beta coefficient of the SNP; SE: standard error; Effect Size: Proportion of the residual phenotypic variance that is explained by the minor allele of the SNP; Gene loc: Gene location; Chr pos: chromosome position in base pairs; *SLC2A9*: solute carrier family 2, member 9; *PPAP2B*: phosphatidic acid phosphatase type 2; *KCNQ1*: voltage gated KQT-like subfamily Q, member 1; *ATP13A5*: ATPase type 13A5; *ABCG2*: ATP-binding cassette family G, member 2; *NUCB*: Nucleobindin.

### Meta-analysis

**Figure 1:**
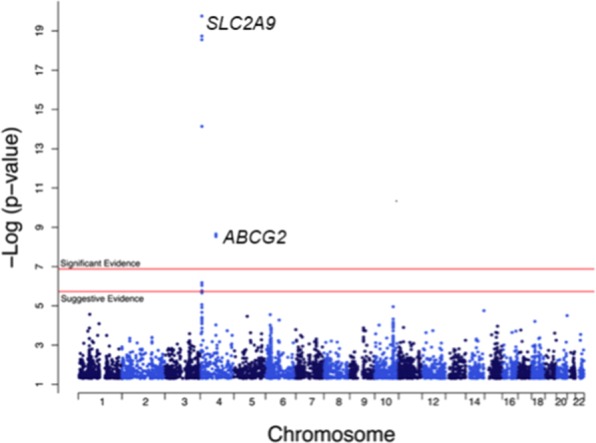
Genome-wide association analysis shows strong association of serum urate with *SLC2A9* and *ABCG2* SNPs.

As a follow-up, we conducted a meta-analysis of SNPs associated with SU concentrations in each of the three centers. The order of the MetaboChip-wide significantly associated SNPs remained similar after meta-analysis, but with increased statistical significance (most significant SNP - rs4481233 (A/G); *P* = 9 × 10^−20^) (Table 3). Individuals with G alleles of rs9998811, rs4148155 and rs1481012 and A alleles of rs7696092, rs4481233, rs13145758 and rs2231142 had lower SU concentrations as compared to other alleles. Table 3 shows these top seven SNPs that exhibited significant associations. Figure 1 shows the Manhattan plot with meta-analysis results showing strong association of *SLC2A9* and *ABCG2* variants on chromosome 4 with SU.

**Table 3:** Variants associated with serum urate in meta-analysis of the three centers.

SNP	Allele1	Allele2	Zscore	*P*-value	Direction	Gene	Gene loc	Chr	Chr pos hg18
rs4481233	A	G	−9.28	1.7 × 10^−20^	—	*SLC2A9*	Intron	4	9956079
rs9998811^*^	A	G	−9.02	1.8 × 10^−19^	—	*SLC2A9*	Intron	4	9966477
rs7696092	A	C	7.78	7.2 × 10^−19^	+++	*SLC2A9*	Intron	4	10025320
rs13145758*	A	G	8.98	2.8 × 10^−14^	+++	*SLC2A9*	Intron	4	9981997
rs4148155^*^	A	G	−5.98	2.2 × 10^−09^	—	*ABCG2*	Intron	4	89054667
rs2231142^*^	A	C	5.94	2.8 × 10^−09^	+++	*ABCG2*	Missense	4	89052323
rs1481012^*^	A	G	−5.94	2.9 × 10^−09^	—	*ABCG2*	Intron	4	89039082

*SNPs are in linkage disequilibrium (LD; r^2^ > 0.90); SNP: single nucleotide polymorphism; P-value: P-values from meta-analysis; Gene loc: Gene location; Chr pos: chromosome position in base pairs; *SLC2A9*: solute carrier family 2, member 9; *ABCG2*: ATP-binding cassette family G, member 2.

### SLC2A9 sequence variants

**Figure 2:**
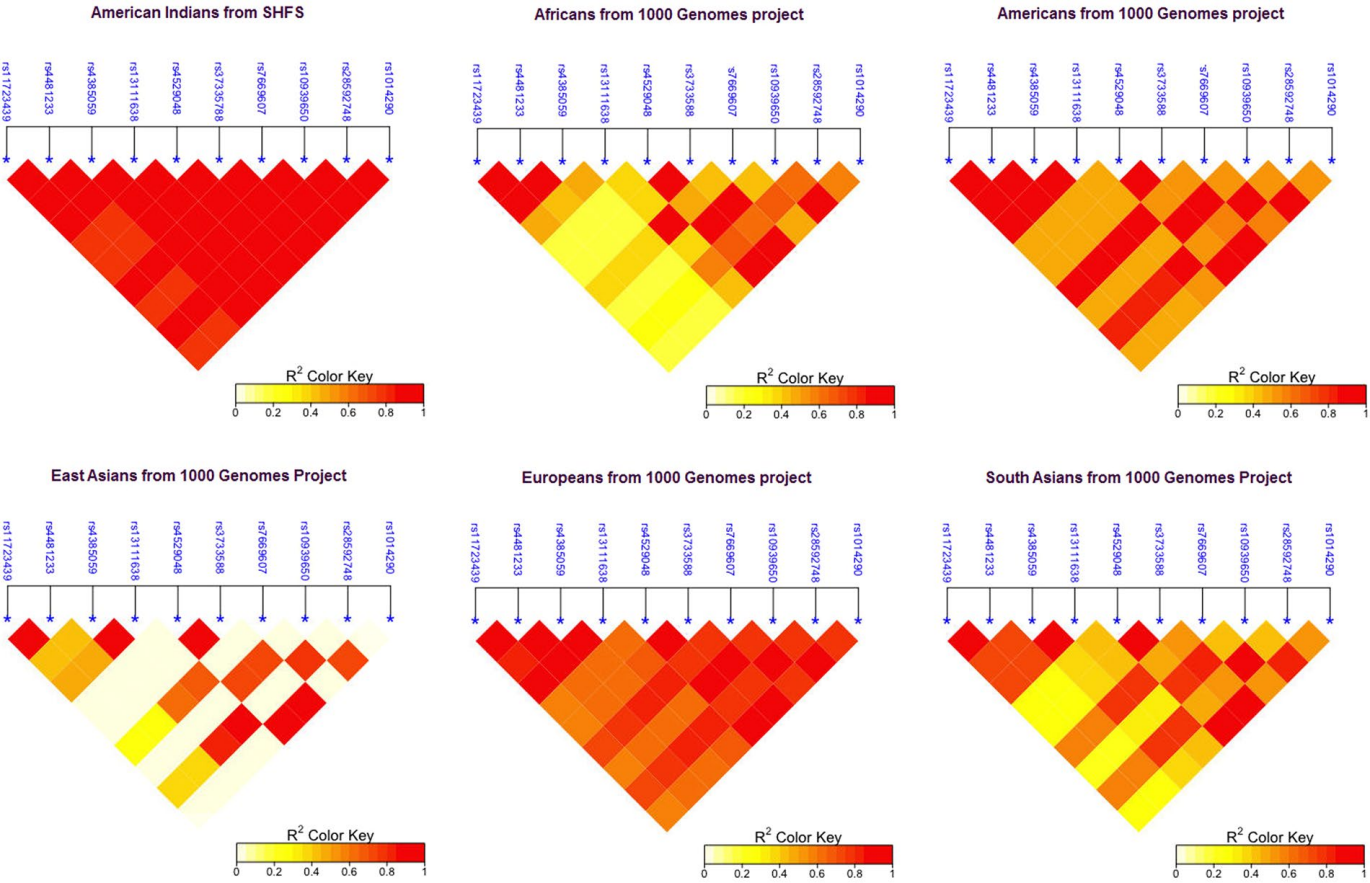
Comparison of LD patterns between ethnicities for top 10SU-associated *SLC2A9* sequence variants.

To identify American Indian-specific SNPs in our top hit, we conducted targeted sequencing of key regions of *SLC2A9* including all exons, 74Kb of introns and 10 kb of upstream and downstream regions of the gene in 902 SHFS founders (individuals with offspring in the SHFS but no parents). A total of 427 autosomal, polymorphic variants were identified in the 96 kb region of *SLC2A9* that was sequenced. These included 233 single nucleotide polymorphisms (SNPs; MAF ≥ 1%); 125 single nucleotide variants (SNVs; minor allele count ≥2); 26 singletons (variants found only in one of our samples); and 43 indels/triallelics (insertions or deletions or more than two alleles); 117 variants of these were novel based on comparison with dbSNP database (Supplementary Table 1). The 233 SNPs were then genotyped in all 3000 SHFS (Arizona = 586, Dakotas = 1208, Oklahoma = 1206) participants of all three centers. A total of 89 SNPs were associated at the significance level of *P* < 2 × 10^−4^ after adjustment for multiple tests. Table 4 lists the top 10 SNPs and their associations with SU concentrations. The rest of the SNPs and their association with SU are shown in Supplementary Table 2. Figure 2 shows LD patterns of these 10SNPs in the SHFS and other ethnicities from the 1000 Genomes project^29^.

**Table 4:** *SLC2A9* sequence variants associated with serum urate (Top 10 significant associations reported here).

SNP*	Chr-pos hg19	Major/Minor Allele	MAF	*P*-value	β	SE	Effect Size
rs4481233	9956079	G/A	0.30	4.2 × 10^−24^	−0.35	0.03	0.05
rs11723439	9951819	G/A	0.28	1.5 × 10^−23^	−0.35	0.03	0.05
rs28592748	9998605	G/A	0.25	2.7 × 10^−22^	−0.35	0.04	0.05
rs7669607	9997801	G/A	0.25	8.2 × 10^−22^	−0.35	0.04	0.05
rs13111638	9996890	G/A	0.23	1.4 × 10^−21^	−0.36	0.04	0.05
rs10939650	9998440	A/G	0.35	4.1 × 10^−21^	−0.31	0.03	0.04
rs4529048	9997112	A/C	0.36	5.0 × 10^−21^	−0.31	0.03	0.04
rs3733588	9997303	A/G	0.35	7.3 × 10^−21^	−0.31	0.03	0.04
rs1014290	10001861	A/G	0.35	7.6 × 10^−21^	−0.31	0.03	0.04
rs4385059	9989233	A/G	0.23	3.6 × 10^−20^	−0.34	0.04	0.04

^*^SNPs after accounting for linkage disequilibrium (LD; r^2^ ≥ 0.80); SNP: single nucleotide polymorphism; Chr pos: chromosome position in base pairs; MAF: minor allele frequency; P-value: P-values from measured genotype analysis; β: beta coefficient of the SNP; SE: standard error; Effect Size: Proportion of the residual phenotypic variance that is explained by the minor allele of the SNP; Gene loc: Gene location.

### Genetic analysis conditional on significant SLC2A9 and ABCG2 variants

To identify secondary signals for loci associated with SUA, we repeated GWAS of SU conditional on the significant *SLC2A9* and *ABCG2* variants in each center^30^. We found suggestive evidence of association of SU concentration with SNPs in nucleobindin (*NUCB1-AS1*) (rs746075, *P* = 2.0 × 10^−6^) in Oklahoma and neuronal PAS-domain containing protein 4 (*NPAS4*) (rs7947391, *P* = 3.2 × 10^−6^) in the Dakotas. No significance or suggestive level of significance was found for SNPs in the Arizona center.

### Replication of SU associations in independent cohorts

To ascertain whether SU-associated SNPs, particularly the novel ones, identified in American Indians are generalizable to other ethnicities, we conducted replications in four different cohorts: Europeans (publicly available data)^15^, Mexicans-mestizos^31^, Indians^32^ and East Asians (publicly available data)^33^. Table 5 shows the SNPs identified in SHFS along with the association results in the other four ethnicities. SNPs rs62293300 and rs4385059 of *SLC2A9* were associated with SU with direction of the effect in American Indians being consistent with Mexican-mestizos. rs4385059, rs28592748 and rs7696092 of *SLC2A9* were associated with SU in all three populations. However, the direction of the effect was same in American Indians, Mexican-mestizos and Indians but different in Europeans and East Asians. The SU associations with *NPAS4* SNP rs7947391 was significantly replicated in the cohort of East Asians with the direction of the effect being same as American Indians and Europeans in contrast to Mexican- Mestizos and Indians. rs746075 of *NUCB1* SNP was not associated with SU in any of the replication cohorts.

**Table 5:** Replication of the associations observed in SHFS.

Gene^a^	SNP^b^	Chr: Chr.pos.hg19	Proxy SNP^c^	American Indian ancestry			Mexican-Mestizo ancestry			European ancestry^d^			Indian ancestry			East Asian ancestry^e^		
				Minor allele (frequency)	β (SE)^f^	P value	Minor allele (frequency)	β (SE)	P value	Minor allele (frequency)	β (SE)	P value	Minor allele (frequency)	β (SE)	P value	Minor allele (frequency)	β (SE)	P value
*PLPP3*	rs6688009	1: 24593576		A (0.01)	1.1 (0.2)	9.1 × 10^−6^	A (0.07)	0.08 (0.06)	0.20	T (0.17)	0.004 (0.007)	0.58				T (0.01)	0.04 (0.02)	0.05
*SLC2A9*	rs62293300	4: 9890359		T (0.01)	0.44 (0.2)	0.052	A (0.03)	0.17 (0.09)	0.07									
*SLC2A9*	NOVEL	4: 9896970		C (0.05)	0.25 (0.12)	0.03												
*SLC2A9*	NOVEL	4: 9897372	rs13136962	A (0.34)	0.15 (0.05)	0.001							G (0.44)	0.07 (0.06)	0.20	C (0.32)	−0.03 (0.004)	7.3 × 10^−11^
*SLC2A9*	NOVEL	4: 9923260		T (0.01)	−0.57 (0.22)	0.008												
*SLC2A9*	NOVEL	4: 9930721		T (0.001)	−1.47 (0.8)	0.049												
*SLC2A9*	NOVEL	4: 9931893		C (0.001)	2.2 (1.1)	0.039												
*SLC2A9*	NOVEL	4: 9986995		C (0.26)	−0.13 (0.06)	0.031												
*SLC2A9*	rs4385059	4: 9989233	rs6449213		−0.34 (0.04)	3.7 × 10^−20^	G (0.22)	−0.48 (0.04)	3.1 × 10^−41^	C (0.18)	0.39 (0.007)	0.000	G (0.18)	− 0.17 (0.07)	0.019	C (0.01)	0.20(0.02)	3.9 × 10^−19^
*SLC2A9*	rs28592748	4: 9998605	rs6449213		−0.36 (0.04)	2.7 × 10^−22^	A (0.24)	−0.48 (0.04)	8.6 × 10^−35^	C (0.18)	0.39 (0.007)	0.000	G (0.18)	− 0.17 (0.07)	0.019	T (0.01)	0.18 (0.02)	7.4 × 10^−16^
*SLC2A9*	rs7696092	4: 10025320	rs4543113	C (0.29)	−0.31 (0.05)	1 × 10^−10^	C (0.26)	−0.44 (0.03)	7.0 × 10^−39^	A (0.40)	0.17 (0.005)	3.7 × 10^−202^	G (0.25)	−0.13 (0.06)	0.034	C (0.01)	0.18 (0.02)	5.1 × 10^−16^
*SLC2A9*	NOVEL	4: 10026105		A (0.001)	2.2 (1.1)	0.037												
*SLC2A9*	NOVEL	4: 10027969		T (0.26)	−0.32 (0.05)	5.4 × 10^−10^												
*SLC2A9*	NOVEL	4: 10030657		G (0.001)	2.12 (1.0)	0.039												
*SLC2A9*	NOVEL	4: 10036877		C (0.0007)	1.12 (0.6)	0.066												
*SLC2A9*	NOVEL	4: 10039575		G (0.012)	0.57 (0.24)	0.02												
*-*	rs7862063	9: 110002036	rs10978764	G (0.41)	0.29 (0.05)	4.5 × 10^−8^	A (0.18)	−0.008 (0.05)	0.86	C (0.40)	0.002 (0.006)	0.72	G (0.43)	0.015 (0.06)	0.79	C (0.35)	0.001 (0.005)	0.75
*KCNQ1*	11_2483882	11: 2483882		A (0.45)	−0.23 (0.05)	3.4 × 10^−6^												
*NPAS4*	rs7947391	11: 66186882		A (0.22)	−0.26 (0.06)	5.6 × 10^−6^	G (0.48)	0.011 (0.04)	0.77	A (0.19)	−0.0009 (0.006)	0.89	A (0.20)	0.057 (0.07)	0.39	A (0.23)	−0.01 (0.005)	0.003
*NUCB1*	rs746075	19: 49416936		A (0.41)	0.22 (0.05)	2.8 × 10^−6^				T (0.42)	0.002 (0.008)	0.80	A (0.37)	−0.106 (0.06)	0.067	A (0.41)	−0.0003 (0.004)	0.95

^a^*PLPP3 or PPAP2B:* Phospholipid phosphatase 3; *SLC2A9:* Solute carrier family 2 member 9; *KCNQ1*: Potassium voltage-gated channel subfamily Q member 1; *NPAS4*: Neuronal PAS domain protein 4;

*NUCB1*: Nucleobindin 1; ^b^SNP: Single nucleotide polymorphism (index SNP); ^c^Proxy SNP: SNP in high LD with the index SNP. Used by replication studies when the original SNP data were not available; ^d^http://metabolomics.helmholtz-muenchen.de/gugc/ and http://useast.ensembl.org/Homo_sapiens/Variation/Explore?r=4:9950721; ^e^Kanai *et al*. 2018; Nature Genet. ^f^β (SE): Beta coefficient (standard error).

## Discussion

Our extensive association analysis using genome-wide as well as candidate gene SNPs in American Indians of the SHFS showed that uric acid transporters *SLC2A9* and *ABCG2* are key genes regulating SU concentrations. Previously, significant heritability was obtained, and linkages were localized for SU concentrations in American Indian participants of the SHFS^27^. Also, our previous candidate gene study replicated 7 *SLC2A9* gene polymorphisms in these participants in all centers combined and when stratified by recruitment center^28^. However, so far no genome-wide analyses for SU have been reported in American Indians. In this regard, our MetaboChip data represents for the first time a detailed genome-wide investigation to identify genetic factors affecting the variation in SU in this population.

The top SNPs from our SU association analysis belonged to gene *SLC2A9* located on chromosome 4 confirming results from previous studies from our and other groups^14–22^. The SNPs rs4481233, rs9998811, rs7696092 and rs3145758 were consistently associated with SU across centers. Our meta-analysis reproduced and strengthened our genome-wide MetaboChip results that were found for all the three study centers separately. In addition to *SLC2A9*, *ABCG2* variants also significantly affected SU concentrations, which further implicates uric acid transporters in the regulation of SU concentrations.

Identification of genetic variants underlying complex traits in minority populations in the US is challenging as they are underrepresented in genetic association studies and databases^23^, particularly Native Americans. Previous studies from our group involving minority populations such as Mexican Americans^19^, Zuni Indians^20^ and Hispanic children^21^ have shown *SLC2A9* to be the key gene affecting SU concentrations. The same has been shown by others in Europeans^14,15^, Asians^16,17^ and African Americans^12,22^. However, the associated variants seem to differ by population. Three recent studies have found significant association of SU with rs2231142 of *ABCG2* and rs7678287 of *SLC2A9* in Mexican-mestizos^31^ and rs2231142 of *ABCG2* and rs3775948 of *SLC2A9* in Indians^32^ and rs7679724 of *SLC2A9* and rs4148155 of *ABCG2* in Japanese individuals^33^. While rs2231142 and rs4148155 of *ABCG2* and rs3775948 were strongly associated with SUA in our study, rs7678287 and rs7679724 were not associated with SU in either individual centers or in the meta-analysis.

Our MetaboChip (center-specific and meta-analysis) association analysis has consistently shown rs4481233 of *SLC2A9* to be strongly associated with SU concentration, further confirmed by our sequence variant analysis. An intronic variant, rs4481233, has been shown to be strongly associated with urate and gout^34–36^. The minor allele (A) of rs4481233 has been shown to be associated with lower concentrations of SU^34^ replicated by our study where SU was decreased by 0.32 mg/dl with every allele of the minor allele. Similar results have also been reported in a GWAS of untargeted serum metabolomics in about 3000 individuals from two large population-based European cohorts where rs4481233 was found to be strongly associated with urate^35,36^. Although intronic, rs4481233 is in high LD with missense variant rs16890979 and is part of a LD block that contains the polymorphic *Alu* elements^37^ and regulatory motifs for Ets- and TCF12-family of transcription factors^38^ with potential for affecting splicing and gene expression.

We also found significant associations of SU with SNPs rs2231142, rs4148155, and rs1481012 (all three in LD) belonging to *ABCG2*, yet another uric acid transporter gene. Notably, rs2231142 is a missense variant of *ABCG2* and is likely functional. This variant has been extensively reported to be associated with SU in several populations including Mexican-mestizos^31^, Mexican Americans^13,39^, European Americans, African Americans, and Asian populations^12–17^, although *SLC22A12* has been shown to be strongest gene to be associated with SU in East Asians^18^. *ABCG2* variants had not only been shown to be associated with SU concentrations, but also with fasting glucose^40^, and different forms of cancer^41,42^, especially rs1481012 associated with decreased risk of colorectal cancer^43^. This gene, however, was not associated with SU in a cohort of Zuni Indians, another American Indian group^20^.

Other SNPs that were linked to variation in SU concentrations were rs7862063, located on chromosome 9, and rs6688009 of phosphatidic acid phosphatase type 2 (*PPAP2B or PLPP3*) gene in the Arizona center. Studies have indicated a role for *PPAP2B* in adipogenesis^44^ and vascular inflammation^45^. It seems to have a protective role in endothelial dysfunction by negatively regulating inflammatory cytokines^45^. However, this association was not shown by our other two centers or replication cohorts. In the Dakotas, we observed marginal association of SU with rs179409 of potassium channel, voltage gated KQT-like subfamily Q, member 1 (*KCNQ1*) gene, which has been shown to associate with gout and hyperuricemia^46,47^. The potential mechanism seems to be through alterations in innate immunity^47,48^, though, these variants and our SNP are about ~3 Mb apart and also not in LD with each other. Therefore, these associations need further investigations. KCNQ1 has been widely reported to be associated with body mass index and other anthropometric measures, and also with type 2 diabetes mellitus in several populations^49–51^.

Furthermore, our conditional analysis identified two novel genes, *NUCB1* and *NPAS4*, to be associated with SU. Conditional analysis is a tool that is used to identify secondary signals that may be otherwise masked by the strong effects of lead SNPs^30^. The *NUCB1* and *NPAS4* SNPs were associated with lower SU concentrations and found only in the Oklahoma and Dakota centers, respectively. Center-specific statistics for these two SNPs show considerable differences in minor allele frequencies between them, rs7947391 of *NPAS4* – 0.05, 0.22, 0.13 and rs746075 of *NUCB1* – 0.31, 0.49, 0.36 in AZ, DK, OK, respectively. NUCB1 is a Golgi-protein with potential role in calcium homeostasis and immunity^52^. It is believed to control protein unfolding in Alzheimer’s disease^53^ and stimulate insulin secretion^54^. NPAS4 is a neuronal transcription factor involved in the regulation of cognitive functions in the brains^55^. Its association with SU in our study was replicated in Japanese individuals of East Asian ancestry^33^. In addition, this study also found a significant expression quantitative trait locus (eQTL) for NPAS4 in monocytes (p = 6 × 10^−10^). The common link between NPAS4 and SU seems to be oxidative stress and inflammation-associated ischemia in the brain^56^. This assumes significance considering the increasing importance of uric acid in cognitive, and neuronal function and its recognition as an important biomarker for Parkinson’s disease^57,58^.

Sequence analysis of key regions of *SLC2A9* identified 384 SNPs/SNVs/singleton variants, 117 variants of these were novel based on comparison with dbSNP database. Several of those SNPs, including rs3775946, rs6826764, rs6823877, rs56239136, rs4697693, rs2240721, rs1107710, and rs7698858 have not been previously reported as affecting SU concentrations. One SNP (chr_pos: 4_10027969) was found to be novel. Although, most studies reported the SNPs in the *SLC2A9* locus to be significantly associated with SU in different populations, *SLC2A9* variants such as rs3733585, rs6855911, rs1014290 and rs12499857 associated with SU in our study have also been linked to Parkinson’s disease^59,60^, type 2 diabetes^61^, anxiety disorders^62^ and nonsyndromic cleft palate^63^. Our results also showed that the minor alleles of most of these SNPs were associated with lower SU concentrations.

There are some limitations of the study. First, the MetaboChip may not be the best option for identifying SNP associations with SU in this population. Secondly, the sample size for GWAS may be only moderate. However, family-based studies, a key strength of this study, are favorable for detection of significant associations and/or gene discoveries as they are homogenous, are robust to the effects of population stratification and have increased power to detect novel associations due to reduced residual variance^64^. Another strength of this study is inclusion of an understudied population with unique genetic and environmental background and availability of extensive covariate information.

In summary, our results replicated known associations of uric acid transporters with SU in a minority population of American Indians and demonstrated potential novel associations of SU with neuronal-related genes which need further investigation.

## Methods

### Study population: strong heart family study (SHFS)

The Strong Heart Family Study (SHFS) is a genetic study of CVD risk in American Indians. Description of the phenotypes, SU measurement techniques, and other related analytical approaches have been detailed elsewhere^27,28^. In short, the SHFS is a multi-center family-based genetic study in American Indian communities from Arizona, the Dakotas, and Oklahoma, which are experiencing extraordinarily high rates of progressive chronic kidney disease (CKD), obesity, diabetes, CVD, and diabetic nephropathy. Approximately 3000 members (including 902 founders) belonging to multigenerational families of Arizona, North and South Dakota (Dakotas), and Oklahoma participated in the study. These individuals aged 14 to 93 years were recruited without regard to disease status in 1998^65,66^. The Indian Health Service Institutional Review Board and the Institutional Review Boards from Texas Biomedical Research Institute and the University of North Carolina at Chapel Hill approved the SHFS protocol and all subjects gave informed consent. For participants under the age of 18 years, informed consent was obtained from their parent or legal guardian. Study design and methods of the SHFS are in accordance with institutional guidelines and have been described previously^65,66^.

### Phenotyping

Blood was collected after an overnight fast. Uric acid concentrations in serum were assayed in the SHFS central laboratory by the uricase and peroxidase method^67^.

### Genotyping

#### MetaboChip data

Blood collected from individuals who were free of diabetes at baseline visit (n = 2000) was used for this study. Cardio-Metabo DNA Analysis BeadChip (Illumina catalog# WG-310-1001 or WG-310-1002) was used for genotyping. The MetaboChip contained 196,725 markers. The original annotation file for the Cardio-Metabo BeadChip is Metabochip_Gene_Annotation. Simwalk2 was used to remove genotyping inconsistencies^68^. Participants were excluded if genotyping call rate was <95% (n = 3). SNPs were excluded if the call rate <98% (n = 0), not autosomal (n = 250), no data after imputation (n = 33,599) or Hardy-Weinberg equilibrium P < 1 × 10^−5^ (n = 20,067). Pairwise correlations (r^2^) between markers were calculated to estimate linkage disequilibrium (LD). The final cleaned, imputed data set includes 162,718 autosomal marker information available for 2000 American Indian participants.

### Sequencing of SLC2A9 gene

We sequenced 96 kb of the *SLC2A9* gene, using a Illumina’s TruSeq Custom Amplicon kit and MiSeq Sequencer, in 902 founders of multigenerational families. The target regions contained all exons, 2.2 kb, 74 kb of introns, and 10 kb of upstream and downstream region of the gene. Illumina generated sequence data (BAM files) were aligned to the Human Genome Reference Sequence version 37.1 (hg19). Variants were called, recalibrated and QC’d using the Genome Analysis Toolkits (GATK v.3.3) Haplotype Caller^69^. Pairwise correlations (r^2^) between markers were calculated to estimate linkage disequilibrium (LD). We identified 427 autosomal, non-monomorphic variants, 384 of which affected a single base (233 single nucleotide polymorphisms (SNPs; MAF ≥ 1%); 125 single nucleotide variants (SNVs; minor allele count ≥ 2); 26 singletons (variants found only in one of our samples)); and 43 were indels/triallelic (insertions or deletions or more than two alleles). Out of the forty-three indel/triallelic vaiants, 14 variants were listed in dbSNP (rs140391260, rs5856025, rs34839464, rs137899691, rs35950306, rs139025036, rs35614040, rs58702202, rs112058434, rs66622652, rs60841869, rs142713311, rs3834235, and rs66943961). The MAFs of all variants ranged between 0.1 and 49%, except for indels/triallelic.

### Statistical analysis

#### Genotype cleaning and population stratification assessment

Genotype frequencies for each SNP were estimated and tested for departures from Hardy-Weinberg equilibrium in the software package, Sequential Oligogenic Linkage Analysis Routines (SOLAR)^70^. Also, we used principal component (PC) scores to model differences in ancestral contributions among study participants for MetaboChip data. PCs were calculated using the unrelated SHFS founders and a subset of 15,158 selected SNPs (r^2^ < 0.1; MAF > 0.05). PCA was performed on a matrix of “doses” (copies of minor allele) for the selected SNPs, using “prcomp” in R. The PC scores were then predicted for all genotyped individuals using the PCA model fit to the founder data^71,72^. While no PC accounted for a large percentage of total variance in genotypes scores, the first four PCs account for substantially more than the rest and were, therefore, included as additional covariates in association analyses.

### Measured genotype analysis (MGA)

The association of SNPs with SU was estimated using a measured genotype analysis (MGA)^73^ executed in SOLAR after accounting for family relationships based on variance components approach. This approach allows us to account for the non-independence among family members. To minimize the problem of non-normality, the SU data were inverse-normal-transformed using SOLAR. All analyses involved adjustment for the covariate effects (see results). The appropriate significance level was determined to be *P* < 4 × 10^−7^ for MetaboChip data, and *P* < 2 × 10^−4^ for sequence data after correcting for multiple tests.

#### METAL

METAL^74^ software was used to perform meta-analysis of GWAS results taken from the three study centers, each study containing individual genome-wide MetaboChip association results for multiple markers.

### Conditional analysis and functional annotation of significant variants

To identify additional independent loci that are associated with SU concentrations, we performed association analysis conditioned on significant *SLC2A9* and *ABCG2* SNPs^30^.

### Replication studies

#### Mexican-mestizos studies

Seven of the SNPs that reached genome-wide suggestive significance in the discovery phase were tested for replication in an independent cohort of Mexican Mestizo individuals (1,061 children and 1,101 adults). Population characteristics, biochemical measurements and genotyping have been previously described. Briefly, genotypes of six SNPs were obtained from a Multi-Ethnic Genotyping Array (MEGA, Illumina, San Diego, CA, USA), while rs7947391 genotypes were imputed using 1000 Genomes Project and Native Mexican individuals as ref. ^31^. Associations with SUA were tested separately in children and adults in a linear mixed model that considered the genetic relatedness matrix as a random effect, while genotype, age, sex and BMI percentile or body mass index (kg/m^2^) were included as fixed effects. Results were meta-analyzed with the inverse variance method^75^.

### Indian diabetes consortium

The study participants included the members of the INdian DIabetes Consortium (INDICO)^76^. Details of the study recruitment and phenotype measurements are given in Giri *et al*.^32^. In short, samples were enrolled in the study by conducting diabetes awareness camp organized in various parts of North India. Prior informed written consent was obtained from the study participants. The study was approved by the Human Ethics Committee of the CSIR-Institute of Genomics and Integrative Biology and the All India Institute of Medical Sciences research Ethics Committee. The study was conducted in accordance with the principles of the Helsinki Declaration. Genotyping was conducted using the Illumina Human 610-quad bead chip array. Association with SU concentrations was tested using linear regression models in PLINK^77^. Sex, age, BMI and first three principal components of genotypes were used as covariates in the model.

### European studies

Replication analysis in Europeans was conducted with publicly available data from Kottgen *et al*.^15^. We used the summary statistics from meta-analysis of serum urate from the article by Kottgen *et al*. The meta-analyses comprised of 14 studies totaling 2,115 cases and 67,259 controls [http://metabolomics.helmholtz-muenchen.de/gugc/, http://useast.ensembl.org/Homo_sapiens/Variation/Explore?r=4:9950721-9951721;source=dbSNP;v=rs1079128;vdb=variation;vf=250145324].

### East asian studies

Replication analysis in East Asians was conducted with publicly available data from Kanai *et al*.^33^. We used the summary statistics from a genome-wide association analysis of serum uric acid in Japanese individuals from Kanai *et al*. The GWAS was conducted for 58 quantitative traits, including serum uric acid, in 162,255 individuals [https://www.ncbi.nlm.nih.gov/pubmed/?term=Kanai+M%2C+2018%2C Nature + Genetics]^78^ This research was supported by the Tailor-Made Medical Treatment Program (the BioBank Japan Project) of the Ministry of Education, Culture, Sports, Science, and Technology (MEXT) and the Japan Agency for Medical Research and Development (AMED)^78^.

## Supplementary information

Association of SU with SLC2A9 sequence variants

## Data Availability

The Strong Heart Study is conducted as a partnership between the American Indian Tribes that are part of the study and the study investigators. All the intellectual property and data generated by this project is administered according to policies from the Tribal Nations, research organizations that are involved in the study, and the NIH. The data is owned by the Tribal Nations, not the study investigators. The study investigators accessed the data used in this manuscript through a formal request for data after a paper proposal was approved by the Strong Heart Study Publication and Presentation committee and following all the procedures that have been approved by the Tribal Nations. The protocols for paper proposal and data access requests can be found on the SHS website: http://strongheart.ouhsc.edu/. The authors confirm that interested researchers may apply for access to these data in the manner described.
