# Supplementary material for: Fine mapping and identification of serum urate loci in American Indians: The Strong Heart Family Study
Source: Sci Rep. 2019 Nov 29;9:17899. doi: 10.1038/s41598-019-52924-w (PMC6884539; doi:10.1038/s41598-019-52924-w)
Supplement: Supplementary file 1 — Association of SU with SLC2A9 sequence variants [file 41598_2019_52924_MOESM1_ESM.docx]

**Fine mapping and identification of serum urate loci in American Indians: The Strong Heart Family Study**

Geetha Chittoor^1,2^, Karin Haack^3^, Poojitha Balakrishnan^4^, Christopher Bizon^5^, Sandra Laston^6^, Lyle G. Best^7^, Jean W. MacCluer^3^, Kari E North^8^, Jason G. Umans^9^, Nora Franceschini^8^, Gauri Prasad,^10,11^ Luis Macias-Kauffer,^12^ Teresa Villarreal-Molina,^12^ Dwaipayan Bharadwaj,^11,14^ Samuel Canizales-Quinteros,^13^ Ana Navas-Acien^4^, Shelley A. Cole^3^, V. Saroja Voruganti*^1^

^1^Department of Nutrition, and UNC Nutrition Research Institute, University of North Carolina at Chapel Hill, NC, USA;

^2^Biomedical and Translational Informatics, Geisinger, Danville, PA, USA;

^3^Population Health Program, Texas Biomedical Research Institute, San Antonio, TX, USA; ^4^Department of Environmental Health Sciences, Columbia University Mailman School of Public Health, New York, New York, USA;

^5^Renaissance Computing Institute, University of North Carolina, Chapel Hill, NC, USA;

^6^South Texas Diabetes and Obesity Institute, School of Medicine, University of Texas Rio Grande Valley, Brownsville, TX, USA;

^7^Missouri Breaks Industries Research Inc., Eagle Butte, SD, USA;

^8^Department of Epidemiology, University of North Carolina at Chapel Hill, NC, USA;

^9^Medstar Health Research Institute, Hyattsville, MD, USA;

^10^Genomics and Molecular Medicine Unit, CSIR-Institute of Genomics and Integrative Biology, New Delhi 110 020, India;

^11^Academy of Scientiﬁc and Innovative Research, CSIR-Institute of Genomics and Integrative Biology Campus, New Delhi-110 020, India:

^12^ Laboratorio de Enfermedades Cardiovasculares, INMEGEN, Mexico City 14610, Mexico.

^13^ Unidad de Genomica de Poblaciones Aplicada a la Salud Facultad de Quimica, UNAM-Instituto Nacional de Medicina Genomica

^14^Systems Genomics Laboratory, School of Biotechnology, Jawaharlal Nehru University, New Delhi 110 067, India;

*Corresponding author:

V. Saroja Voruganti, PhD

Assistant Professor

Department of Nutrition and UNC Nutrition Research Institute

500 Laureate Way, suite 3150

Kannapolis, NC 28081

Ph: 704-250-5009

Email: saroja@unc.edu

**SUPPLEMENTARY FILE:** Association of SU with *SLC2A9* sequence variants

- **Supplementary Table 1:** Association of SU with *SLC2A9* locus with novel SNPs identified in American Indian participants
- **Supplementary Table 2:** Association of SU with *SLC2A9* locus with all identified SNPs (other than the novel ones) in American Indian participants
- **Supplementary Table 3:** Association of *SLC2A9* sequence variants with SU in each of the three centers of SHFS. Associations that are significant at p < 2 x10^-4^ in at least one center are shown below

**Supplementary Table 1. Association of SU with *SLC2A9* locus with novel SNPs in American Indian participants**

| **Chr pos hg19** | **Minor Allele** | **MAF** | **P-value** | **β** | **SE** |
| --- | --- | --- | --- | --- | --- |
| 10027969 | T | 0.2558 | 5.44E-10 | -0.3228 | 0.0520 |
| 9897372 | A | 0.3396 | 0.0012 | 0.1542 | 0.0476 |
| 9923260 | T | 0.0111 | 0.007836 | -0.5731 | 0.2155 |
| 10039575 | G | 0.0119 | 0.020496 | 0.5669 | 0.2447 |
| 9986995 | C | 0.2613 | 0.030588 | -0.1317 | 0.0609 |
| 9896970 | C | 0.0448 | 0.030766 | 0.2515 | 0.1164 |
| 10026105 | A | 0.0007 | 0.036786 | 2.2031 | 1.0551 |
| 9931893 | C | 0.0007 | 0.038641 | 2.1819 | 1.0551 |
| 10030657 | G | 0.0007 | 0.039496 | 2.1190 | 1.0292 |
| 9930721 | T | 0.0014 | 0.049675 | -1.4656 | 0.7467 |
| 9890359 | T | 0.0098 | 0.05166 | 0.4393 | 0.2257 |
| 10036877 | C | 0.0007 | 0.065958 | 1.1158 | 0.6069 |
| 9954799 | A | 0.0014 | 0.073706 | -0.9126 | 0.5103 |
| 9950811 | C | 0.0014 | 0.073815 | -0.9126 | 0.5105 |
| 9938284 | A | 0.0061 | 0.106768 | 0.4201 | 0.2604 |
| 9890340 | G | 0.0015 | 0.115483 | -1.1167 | 0.7095 |
| 9926525 | C | 0.0014 | 0.120233 | 0.9364 | 0.6027 |
| 10036938 | T | 0.0014 | 0.12254 | -0.8063 | 0.5222 |
| 9940625 | G | 0.0076 | 0.124901 | -0.4296 | 0.2800 |
| 10036863 | C | 0.0300 | 0.147019 | 0.2126 | 0.1466 |
| 9939239 | A | 0.0246 | 0.170838 | 0.1902 | 0.1389 |
| 9891416 | C | 0.1272 | 0.179324 | -0.0962 | 0.0716 |
| 9894207 | T | 0.0021 | 0.18735 | 0.5846 | 0.4434 |
| 10038244 | C | 0.0035 | 0.192612 | -0.3651 | 0.2802 |
| 10020551 | G | 0.0440 | 0.198195 | -0.1497 | 0.1164 |
| 9990575 | C | 0.0007 | 0.199899 | 1.3316 | 1.0388 |
| 9894996 | G | 0.0129 | 0.206738 | -0.2680 | 0.2123 |
| 9948046 | C | 0.0112 | 0.21254 | 0.2985 | 0.2395 |
| 9992337 | C | 0.0007 | 0.214798 | 1.3098 | 1.0559 |
| 9946857 | C | 0.2901 | 0.233708 | -0.0733 | 0.0615 |
| 10039916 | G | 0.0119 | 0.235608 | 0.3671 | 0.3095 |
| 9889911 | G | 0.0358 | 0.241059 | -0.1731 | 0.1477 |
| 9891856 | C | 0.0007 | 0.243129 | -1.2155 | 1.0414 |
| 9948065 | G | 0.0030 | 0.243975 | 0.5665 | 0.4863 |
| 10030015 | T | 0.0096 | 0.265657 | 0.2710 | 0.2434 |
| 9991237 | C | 0.0111 | 0.265684 | 0.2747 | 0.2468 |
| 9893937 | T | 0.0014 | 0.27014 | -0.5872 | 0.5325 |
| 9948053 | G | 0.0082 | 0.272709 | -0.2614 | 0.2384 |
| 9926459 | C | 0.0249 | 0.287795 | 0.1759 | 0.1655 |
| 9995019 | C | 0.0042 | 0.292525 | 0.3558 | 0.3381 |
| 9940810 | C | 0.2024 | 0.301496 | 0.0636 | 0.0615 |
| 9922527 | C | 0.0103 | 0.304208 | 0.2596 | 0.2527 |
| 9894573 | G | 0.0021 | 0.310498 | 0.4740 | 0.4674 |
| 10038481 | G | 0.0007 | 0.313325 | -1.0647 | 1.0560 |
| 9950815 | T | 0.0014 | 0.319044 | 0.5538 | 0.5558 |
| 10027496 | C | 0.1477 | 0.324298 | -0.0657 | 0.0667 |
| 9898999 | G | 0.0007 | 0.325039 | -1.0254 | 1.0419 |
| 9896601 | C | 0.0035 | 0.349153 | 0.3762 | 0.4018 |
| 10042945 | C | 0.0199 | 0.381995 | -0.1566 | 0.1791 |
| 10038338 | C | 0.0094 | 0.385223 | 0.2183 | 0.2514 |
| 10038339 | T | 0.0094 | 0.385223 | 0.2183 | 0.2514 |
| 10038343 | T | 0.0094 | 0.385223 | 0.2183 | 0.2514 |
| 10038347 | A | 0.0094 | 0.385223 | 0.2183 | 0.2514 |
| 10038355 | G | 0.0094 | 0.385223 | 0.2183 | 0.2514 |
| 10039339 | G | 0.0095 | 0.403862 | 0.2065 | 0.2474 |
| 9995070 | C | 0.2143 | 0.40782 | -0.0507 | 0.0613 |
| 9947290 | G | 0.0048 | 0.418674 | 0.3156 | 0.3903 |
| 9889686 | G | 0.0099 | 0.422619 | -0.2152 | 0.2683 |
| 9948060 | C | 0.0090 | 0.427895 | 0.2102 | 0.2651 |
| 9927014 | T | 0.0035 | 0.44866 | -0.3492 | 0.4609 |
| 10038747 | T | 0.0035 | 0.44866 | -0.3492 | 0.4609 |
| 9889900 | C | 0.0397 | 0.474534 | -0.0926 | 0.1295 |
| 9889700 | C | 0.0086 | 0.481182 | 0.1785 | 0.2534 |
| 9827662 | C | 0.0007 | 0.487924 | 0.6987 | 1.0073 |
| 9827564 | A | 0.0178 | 0.490038 | -0.1074 | 0.1555 |
| 10036671 | T | 0.0021 | 0.519108 | -0.2510 | 0.3894 |
| 9898626 | C | 0.0523 | 0.526403 | 0.0718 | 0.1134 |
| 10039268 | C | 0.0063 | 0.56425 | 0.1915 | 0.3321 |
| 9891820 | C | 0.0007 | 0.565687 | -0.6067 | 1.0562 |
| 9927012 | C | 0.2041 | 0.566749 | 0.0359 | 0.0627 |
| 9948292 | C | 0.0021 | 0.572037 | 0.3318 | 0.5872 |
| 9996724 | C | 0.1489 | 0.584248 | 0.0372 | 0.0680 |
| 9894178 | C | 0.2789 | 0.591971 | 0.0324 | 0.0604 |
| 10038951 | T | 0.0007 | 0.606434 | 0.4268 | 0.8284 |
| 9890519 | C | 0.0028 | 0.612904 | -0.2294 | 0.4534 |
| 9931141 | T | 0.0172 | 0.635887 | -0.0803 | 0.1696 |
| 9948064 | G | 0.0097 | 0.64116 | -0.1232 | 0.2643 |
| 9891963 | C | 0.0014 | 0.668393 | 0.2372 | 0.5538 |
| 9893627 | C | 0.0879 | 0.683657 | 0.0344 | 0.0844 |
| 10038169 | C | 0.0007 | 0.695605 | -0.4133 | 1.0562 |
| 9948134 | C | 0.0095 | 0.71421 | -0.0923 | 0.2521 |
| 9929216 | C | 0.0014 | 0.714547 | 0.2734 | 0.7474 |
| 9927163 | T | 0.0007 | 0.720366 | 0.2283 | 0.6378 |
| 9889612 | C | 0.0138 | 0.727062 | -0.0859 | 0.2461 |
| 10039353 | G | 0.0141 | 0.736532 | -0.0720 | 0.2139 |
| 9992331 | C | 0.0021 | 0.736803 | -0.1892 | 0.5628 |
| 9926492 | C | 0.0007 | 0.737505 | 0.3540 | 1.0562 |
| 9991784 | C | 0.0619 | 0.739578 | 0.0336 | 0.1009 |
| 9939301 | G | 0.0050 | 0.744668 | -0.0957 | 0.2939 |
| 9929303 | T | 0.0007 | 0.749436 | -0.3329 | 1.0425 |
| 9946021 | G | 0.0007 | 0.749436 | -0.3329 | 1.0425 |
| 9938545 | C | 0.0032 | 0.75524 | -0.1342 | 0.4306 |
| 9992874 | C | 0.0309 | 0.758409 | 0.0446 | 0.1451 |
| 10039908 | G | 0.0257 | 0.768112 | -0.0611 | 0.2072 |
| 10038680 | T | 0.0007 | 0.773387 | 0.2696 | 0.9363 |
| 9889886 | C | 0.0452 | 0.782612 | -0.0354 | 0.1284 |
| 9889638 | G | 0.0060 | 0.793472 | -0.0994 | 0.3797 |
| 9990135 | C | 0.1343 | 0.795944 | 0.0182 | 0.0703 |
| 9991532 | A | 0.0014 | 0.799882 | -0.1487 | 0.5866 |
| 9896114 | A | 0.0158 | 0.80234 | -0.0435 | 0.1739 |
| 9891562 | C | 0.0014 | 0.81621 | 0.1672 | 0.7195 |
| 10039280 | G | 0.0091 | 0.828246 | 0.0600 | 0.2765 |
| 9991134 | T | 0.0021 | 0.83034 | -0.1234 | 0.5760 |
| 10039443 | T | 0.0021 | 0.833586 | 0.0998 | 0.4748 |
| 9889856 | T | 0.0162 | 0.848819 | -0.0352 | 0.1847 |
| 9935553 | C | 0.0007 | 0.867396 | -0.1703 | 1.0198 |
| 10038520 | C | 0.1216 | 0.873914 | -0.0115 | 0.0727 |
| 9950790 | C | 0.4267 | 0.885902 | 0.0124 | 0.0867 |
| 9898513 | C | 0.0062 | 0.890332 | -0.0412 | 0.2988 |
| 9996089 | A | 0.0062 | 0.890332 | -0.0412 | 0.2988 |
| 9950966 | G | 0.0007 | 0.927044 | -0.0941 | 1.0282 |
| 10033896 | G | 0.0007 | 0.927044 | -0.0941 | 1.0282 |
| 9953630 | C | 0.0333 | 0.956131 | 0.0067 | 0.1221 |
| 9897522 | C | 0.0652 | 0.980786 | -0.0023 | 0.0968 |
| 10020397 | C | 0.0035 | 0.983189 | 0.0091 | 0.4304 |
| 9891492 | G | 0.0007 | 0.988887 | 0.0110 | 0.7872 |

SNP: single nucleotide polymorphism; Chr pos: chromosome position in base pairs; MAF: minor allele frequency; P-value: P-values from measured genotype analysis; β: beta coefficient of the SNP; SE: standard error; Effect Size: Proportion of the residual phenotypic variance that is explained by the minor allele of the SNP

**Supplementary Table 2. Association of SU with *SLC2A9* locus with all identified SNPs (other than novel ones) in American Indian participants**

| **SNP** | **Chr pos hg19** | **Minor Allele** | **MAF** | **P-value** | **β** | **SE** |
| --- | --- | --- | --- | --- | --- | --- |
| rs3775946 | 9995256 | A | 0.4191 | 5.19E-12 | -0.3208 | 0.0465 |
| rs7663032 | 9993838 | C | 0.4203 | 5.38E-12 | -0.3211 | 0.0466 |
| rs3775948 | 9995182 | G | 0.4215 | 5.59E-12 | -0.3212 | 0.0466 |
| rs10006397 | 10036140 | C | 0.3807 | 1.94E-11 | -0.3064 | 0.0457 |
| rs6826764 | 10030794 | G | 0.3807 | 1.94E-11 | -0.3064 | 0.0457 |
| rs7696983 | 9995829 | A | 0.2860 | 2.28E-11 | -0.3412 | 0.0510 |
| rs4428284 | 9996392 | A | 0.2786 | 1.20E-10 | -0.3286 | 0.0510 |
| rs13111638 | 9996890 | T | 0.2796 | 1.22E-10 | -0.3288 | 0.0511 |
| rs4697701 | 9946095 | G | 0.3811 | 1.48E-10 | 0.3018 | 0.0471 |
| rs4447862 | 9931645 | C | 0.3843 | 1.60E-10 | 0.2999 | 0.0469 |
| rs6449137 | 9932479 | A | 0.3843 | 1.60E-10 | 0.2999 | 0.0469 |
| rs938558 | 9939205 | A | 0.3843 | 1.60E-10 | 0.2999 | 0.0469 |
| rs13129697 | 9926967 | T | 0.3820 | 6.21E-10 | 0.2904 | 0.0470 |
| rs17187075 | 9990328 | G | 0.4768 | 1.10E-09 | -0.2882 | 0.0473 |
| rs7678012 | 9993772 | T | 0.4770 | 1.10E-09 | -0.2878 | 0.0472 |
| rs7669090 | 10028131 | C | 0.3678 | 1.97E-09 | -0.2790 | 0.0465 |
| rs3733584 | 10036506 | T | 0.4542 | 2.59E-09 | -0.2763 | 0.0464 |
| rs3733585 | 10036339 | A | 0.4542 | 2.59E-09 | -0.2763 | 0.0464 |
| rs12499857 | 9995376 | G | 0.3802 | 3.19E-09 | -0.2875 | 0.0486 |
| rs11723382 | 9954660 | T | 0.3301 | 5.30E-09 | 0.2896 | 0.0496 |
| rs7375599 | 9954918 | A | 0.3301 | 5.30E-09 | 0.2896 | 0.0496 |
| rs10939620 | 9946132 | C | 0.3295 | 5.57E-09 | 0.2892 | 0.0496 |
| rs144651801 | 9947865 | T | 0.3321 | 1.04E-08 | 0.2819 | 0.0492 |
| rs1122141 | 9947278 | T | 0.3328 | 1.07E-08 | 0.2816 | 0.0492 |
| rs734553 | 9923004 | G | 0.4809 | 1.11E-08 | -0.2594 | 0.0454 |
| rs2240720 | 10020480 | C | 0.4460 | 1.33E-08 | -0.2628 | 0.0463 |
| rs4447863 | 9938969 | T | 0.3251 | 1.82E-08 | 0.2793 | 0.0496 |
| rs13139055 | 10038924 | G | 0.3406 | 2.02E-08 | -0.2749 | 0.0490 |
| rs12506122 | 10033538 | C | 0.3399 | 2.52E-08 | -0.2736 | 0.0491 |
| rs938564 | 9922573 | G | 0.4759 | 2.83E-08 | -0.2511 | 0.0452 |
| rs13124563 | 9927553 | G | 0.3277 | 4.72E-08 | 0.2726 | 0.0499 |
| rs16890979 | 9922167 | T | 0.4738 | 4.85E-08 | -0.2470 | 0.0453 |
| rs938555 | 9926051 | A | 0.4738 | 4.85E-08 | -0.2470 | 0.0453 |
| rs2240722 | 10020757 | A | 0.4407 | 5.51E-08 | -0.2564 | 0.0472 |
| rs34709913 | 10028955 | T | 0.4437 | 5.96E-08 | -0.2541 | 0.0469 |
| rs55959894 | 10026580 | G | 0.4437 | 5.96E-08 | -0.2541 | 0.0469 |
| rs6823324 | 9932359 | C | 0.4905 | 6.11E-08 | -0.2440 | 0.0450 |
| rs7670751 | 9938773 | C | 0.4905 | 6.11E-08 | -0.2440 | 0.0450 |
| rs34501273 | 9996996 | C | 0.4441 | 6.69E-08 | -0.2550 | 0.0472 |
| rs3796842 | 9995851 | A | 0.4441 | 6.69E-08 | -0.2550 | 0.0472 |
| rs3756237 | 10013378 | G | 0.4450 | 6.99E-08 | -0.2516 | 0.0467 |
| rs10017945 | 9937852 | G | 0.4910 | 7.30E-08 | -0.2426 | 0.0451 |
| rs6855911 | 9935910 | G | 0.4910 | 7.30E-08 | -0.2426 | 0.0451 |
| rs7349721 | 10042562 | T | 0.3242 | 8.15E-08 | 0.3702 | 0.0690 |
| rs3756236 | 10013463 | T | 0.4429 | 9.14E-08 | -0.2505 | 0.0469 |
| rs6838021 | 9927620 | T | 0.4858 | 9.17E-08 | -0.2398 | 0.0449 |
| rs3756238 | 10013202 | C | 0.4446 | 1.16E-07 | -0.2483 | 0.0469 |
| rs4475146 | 9946656 | A | 0.4850 | 1.33E-07 | -0.2365 | 0.0448 |
| rs4280729 | 10013861 | C | 0.4385 | 1.73E-07 | -0.2454 | 0.0470 |
| rs6837336 | 9932510 | A | 0.4839 | 2.89E-07 | -0.2304 | 0.0449 |
| rs3775947 | 9995240 | T | 0.3994 | 1.80E-06 | 0.3906 | 0.0818 |
| rs13137074 | 10042842 | T | 0.2947 | 2.66E-06 | 0.3159 | 0.0673 |
| rs6823877 | 9921931 | T | 0.2381 | 2.68E-06 | 0.2592 | 0.0552 |
| rs13101785 | 10042915 | A | 0.3874 | 2.71E-06 | 0.2203 | 0.0469 |
| rs7679916 | 10042160 | T | 0.3874 | 2.71E-06 | 0.2203 | 0.0469 |
| rs11727199 | 10036190 | T | 0.2955 | 3.62E-06 | 0.3118 | 0.0673 |
| rs7694997 | 9947811 | A | 0.4616 | 4.39E-06 | 0.2088 | 0.0455 |
| rs7375587 | 9954758 | A | 0.4614 | 4.74E-06 | 0.2082 | 0.0455 |
| rs1122142 | 9947548 | G | 0.4591 | 4.85E-06 | 0.2078 | 0.0455 |
| rs4339211 | 9947658 | C | 0.4591 | 4.85E-06 | 0.2078 | 0.0455 |
| rs4311316 | 9955971 | C | 0.4615 | 5.65E-06 | 0.2063 | 0.0455 |
| rs4519796 | 9955936 | A | 0.4615 | 5.65E-06 | 0.2063 | 0.0455 |
| rs12498956 | 9950705 | C | 0.4610 | 6.07E-06 | 0.2058 | 0.0455 |
| rs12498150 | 9950537 | C | 0.4602 | 6.13E-06 | 0.2056 | 0.0455 |
| rs9994266 | 9954450 | G | 0.4628 | 6.95E-06 | 0.2047 | 0.0455 |
| rs4459990 | 9954005 | T | 0.4636 | 8.40E-06 | 0.2029 | 0.0455 |
| rs4292332 | 9953830 | C | 0.4629 | 8.77E-06 | 0.2023 | 0.0455 |
| rs4292333 | 9953871 | G | 0.4629 | 8.77E-06 | 0.2023 | 0.0455 |
| rs4407505 | 9953367 | T | 0.4629 | 8.77E-06 | 0.2023 | 0.0455 |
| rs4447861 | 9953940 | C | 0.4629 | 8.77E-06 | 0.2023 | 0.0455 |
| rs4467563 | 9953460 | A | 0.4629 | 8.77E-06 | 0.2023 | 0.0455 |
| rs4467564 | 9953472 | G | 0.4629 | 8.77E-06 | 0.2023 | 0.0455 |
| rs4560411 | 9953361 | A | 0.4629 | 8.77E-06 | 0.2023 | 0.0455 |
| rs7378305 | 9954893 | C | 0.4629 | 8.77E-06 | 0.2023 | 0.0455 |
| rs4276278 | 9948870 | T | 0.4606 | 8.96E-06 | 0.2019 | 0.0455 |
| rs4408959 | 9948868 | C | 0.4606 | 8.96E-06 | 0.2019 | 0.0455 |
| rs4455410 | 9953297 | T | 0.4633 | 0.000013 | 0.1983 | 0.0455 |
| rs7376948 | 9954708 | G | 0.4633 | 0.000013 | 0.1983 | 0.0455 |
| rs56239136 | 9898003 | C | 0.3342 | 0.000014 | -0.2106 | 0.0485 |
| rs4697694 | 9896642 | C | 0.3393 | 0.000016 | -0.2092 | 0.0485 |
| rs7377578 | 9952775 | T | 0.4668 | 0.000017 | 0.1940 | 0.0451 |
| rs7376505 | 9952744 | C | 0.4682 | 0.000017 | 0.1939 | 0.0450 |
| rs4697693 | 9895860 | C | 0.3401 | 0.000021 | -0.2065 | 0.0485 |
| rs56038393 | 9897885 | A | 0.3378 | 0.00003 | -0.2023 | 0.0485 |
| rs56113653 | 9898052 | G | 0.2761 | 0.000041 | -0.2679 | 0.0653 |
| rs12506932 | 10039528 | A | 0.3912 | 0.000044 | 0.1864 | 0.0456 |
| rs998676 | 9948564 | C | 0.2635 | 0.000051 | -0.2623 | 0.0647 |
| rs10939605 | 9896293 | A | 0.2761 | 0.000061 | -0.2622 | 0.0654 |
| rs938554 | 9925692 | G | 0.3274 | 0.000095 | 0.1416 | 0.0363 |
| rs2240721 | 10020564 | G | 0.3620 | 0.000109 | -0.1842 | 0.0476 |
| rs1107710 | 9890708 | A | 0.4238 | 0.000115 | 0.1779 | 0.0461 |
| rs5028843 | 9940806 | A | 0.3568 | 0.000132 | -0.2791 | 0.0730 |
| rs12644047 | 9893403 | G | 0.4264 | 0.000157 | 0.1744 | 0.0461 |
| rs7698858 | 9895070 | C | 0.4893 | 0.000188 | -0.1578 | 0.0423 |
| rs4697692 | 9893197 | C | 0.4326 | 0.000250 | 0.1685 | 0.0460 |
| rs938563 | 9890998 | G | 0.3439 | 0.000511 | 0.1690 | 0.0486 |
| rs4580649 | 9948461 | A | 0.3887 | 0.001046 | -0.2436 | 0.0743 |
| rs13107656 | 9896938 | G | 0.3396 | 0.0012 | 0.1542 | 0.0476 |
| rs13115121 | 9897342 | G | 0.3396 | 0.0012 | 0.1542 | 0.0476 |
| rs6449097 | 9896734 | C | 0.3396 | 0.0012 | 0.1542 | 0.0476 |
| rs13122026 | 9898207 | C | 0.3403 | 0.001288 | 0.1530 | 0.0475 |
| rs998675 | 9948829 | T | 0.4341 | 0.001435 | -0.3135 | 0.0984 |
| rs4621429 | 9826870 | G | 0.3497 | 0.00168 | -0.1782 | 0.0567 |
| rs6822889 | 9826757 | C | 0.4955 | 0.001799 | -0.1470 | 0.0471 |
| rs13114702 | 9895176 | C | 0.3537 | 0.002013 | 0.1476 | 0.0478 |
| rs6845554 | 10013173 | G | 0.4401 | 0.002108 | 0.3141 | 0.1022 |
| rs2018643 | 9947121 | C | 0.3856 | 0.002257 | -0.2355 | 0.0771 |
| rs10939602 | 9892102 | T | 0.3201 | 0.002275 | 0.1508 | 0.0494 |
| rs7686538 | 9948077 | C | 0.4737 | 0.0026 | 0.1230 | 0.0408 |
| rs12506455 | 10031569 | A | 0.1047 | 0.002809 | 0.2403 | 0.0804 |
| rs4697908 | 9893282 | T | 0.3220 | 0.003901 | 0.1424 | 0.0494 |
| rs7669444 | 9893577 | A | 0.3220 | 0.003901 | 0.1424 | 0.0494 |
| rs115764731 | 9992179 | A | 0.0028 | 0.005155 | 1.4480 | 0.5177 |
| rs76574510 | 9953252 | A | 0.1474 | 0.006294 | -0.1906 | 0.0698 |
| rs4621431 | 9947590 | A | 0.3804 | 0.013159 | -0.1873 | 0.0755 |
| rs6815895 | 9923165 | T | 0.0491 | 0.013701 | 0.2865 | 0.1162 |
| rs28480661 | 9937798 | A | 0.0551 | 0.016634 | 0.2590 | 0.1081 |
| rs62296002 | 9992862 | T | 0.0083 | 0.018854 | 0.6009 | 0.2559 |
| rs938562 | 9891031 | T | 0.3959 | 0.019506 | -0.1943 | 0.0832 |
| rs4511996 | 9939818 | A | 0.0556 | 0.020696 | 0.2489 | 0.1076 |
| rs146808983 | 10039331 | A | 0.0486 | 0.023723 | 0.2608 | 0.1153 |
| rs73229828 | 10026833 | G | 0.0452 | 0.025209 | 0.2663 | 0.1190 |
| rs9992406 | 9986288 | T | 0.0537 | 0.046887 | 0.2163 | 0.1089 |
| rs16891234 | 9946163 | C | 0.0557 | 0.054083 | 0.2048 | 0.1063 |
| rs55962381 | 9898764 | T | 0.0346 | 0.054651 | 0.2546 | 0.1325 |
| rs9998739 | 9996509 | A | 0.0558 | 0.055328 | 0.2035 | 0.1062 |
| rs10011206 | 9991955 | T | 0.0527 | 0.063313 | 0.2118 | 0.1140 |
| rs115768496 | 9953991 | C | 0.0007 | 0.071552 | -1.8082 | 1.0035 |
| rs75815764 | 9827738 | C | 0.0007 | 0.071552 | -1.8082 | 1.0035 |
| rs62293301 | 9891488 | A | 0.0243 | 0.07207 | 0.2975 | 0.1654 |
| rs116692722 | 9926357 | C | 0.0007 | 0.074244 | -1.1449 | 0.6414 |
| rs79687993 | 9896742 | A | 0.0007 | 0.074244 | -1.1449 | 0.6414 |
| rs34325511 | 10028867 | A | 0.3185 | 0.075445 | 0.0895 | 0.0504 |
| rs73806483 | 10036932 | T | 0.0014 | 0.078759 | -0.8926 | 0.5078 |
| rs55921149 | 9993743 | T | 0.0069 | 0.080734 | -0.5322 | 0.3048 |
| rs185415458 | 9940428 | T | 0.0007 | 0.082764 | -1.8311 | 1.0554 |
| rs150084622 | 9993638 | T | 0.0306 | 0.083399 | -0.2147 | 0.1240 |
| rs182760630 | 9946816 | C | 0.0306 | 0.083399 | -0.2147 | 0.1240 |
| rs185455331 | 9890913 | A | 0.0063 | 0.084501 | 0.4819 | 0.2794 |
| rs377337620 | 9993028 | A | 0.0007 | 0.085916 | 1.8134 | 1.0560 |
| rs6449237 | 10027643 | G | 0.0724 | 0.105927 | 0.1505 | 0.0931 |
| rs6449238 | 10027744 | A | 0.0724 | 0.105927 | 0.1505 | 0.0931 |
| rs6819833 | 10027354 | G | 0.0724 | 0.105927 | 0.1505 | 0.0931 |
| rs6820230 | 10027542 | T | 0.0724 | 0.105927 | 0.1505 | 0.0931 |
| rs7697004 | 10028077 | A | 0.0724 | 0.105927 | 0.1505 | 0.0931 |
| rs9291645 | 10038254 | A | 0.0724 | 0.105927 | 0.1505 | 0.0931 |
| rs6858614 | 9889740 | T | 0.3113 | 0.108812 | 0.0779 | 0.0486 |
| rs6836200 | 9986915 | C | 0.1343 | 0.116563 | -0.1030 | 0.0656 |
| rs6849736 | 9986783 | A | 0.1343 | 0.116563 | -0.1030 | 0.0656 |
| rs6849962 | 9986903 | A | 0.1343 | 0.116563 | -0.1030 | 0.0656 |
| rs73100621 | 9986339 | G | 0.1343 | 0.116563 | -0.1030 | 0.0656 |
| rs10030570 | 10027160 | T | 0.0678 | 0.117275 | 0.1583 | 0.1011 |
| rs12646380 | 9991502 | C | 0.1128 | 0.119091 | -0.1174 | 0.0753 |
| rs76289526 | 9940468 | T | 0.0014 | 0.121469 | -0.8496 | 0.5486 |
| rs77071180 | 9935177 | A | 0.0014 | 0.121469 | -0.8496 | 0.5486 |
| rs118123197 | 9940970 | C | 0.0014 | 0.12254 | -0.8063 | 0.5222 |
| rs73225835 | 9890535 | T | 0.0223 | 0.123939 | 0.2614 | 0.1699 |
| rs4502681 | 9990172 | C | 0.1355 | 0.130717 | -0.0988 | 0.0654 |
| rs114056737 | 9991519 | T | 0.0061 | 0.135796 | 0.4863 | 0.3260 |
| rs150665766 | 9990467 | T | 0.0007 | 0.140868 | 0.9774 | 0.6637 |
| rs141099164 | 9893721 | A | 0.0007 | 0.175161 | -1.4314 | 1.0558 |
| rs190346690 | 9991901 | T | 0.1690 | 0.183803 | 0.0822 | 0.0619 |
| rs148657180 | 9939649 | A | 0.0126 | 0.185281 | 0.2534 | 0.1913 |
| rs77870747 | 9993636 | C | 0.0229 | 0.194107 | 0.2071 | 0.1595 |
| rs68013308 | 9992672 | A | 0.1280 | 0.200586 | -0.0855 | 0.0668 |
| rs143241420 | 9891611 | A | 0.0007 | 0.208218 | 1.0383 | 0.8250 |
| rs186263967 | 9995002 | T | 0.1638 | 0.216707 | 0.0772 | 0.0625 |
| rs112430423 | 10026036 | C | 0.0035 | 0.219553 | 0.5771 | 0.4701 |
| rs6820616 | 10030076 | A | 0.1082 | 0.222202 | -0.0868 | 0.0711 |
| rs147439739 | 9895642 | C | 0.0035 | 0.23736 | 0.5002 | 0.4234 |
| rs13116337 | 9947669 | A | 0.1123 | 0.244542 | -0.0884 | 0.0759 |
| rs4467565 | 9953487 | A | 0.1123 | 0.244542 | -0.0884 | 0.0759 |
| rs59234705 | 10026745 | C | 0.1111 | 0.261219 | -0.0792 | 0.0705 |
| rs143885121 | 9927648 | T | 0.0014 | 0.261537 | -0.5731 | 0.5105 |
| rs144428359 | 9828099 | A | 0.0014 | 0.261537 | -0.5731 | 0.5105 |
| rs66647240 | 9990658 | A | 0.1263 | 0.272773 | -0.0736 | 0.0671 |
| rs77684901 | 10026937 | T | 0.0035 | 0.276911 | 0.4879 | 0.4488 |
| rs73229836 | 10033933 | T | 0.0207 | 0.282373 | 0.1844 | 0.1715 |
| rs80204783 | 9894371 | T | 0.0069 | 0.287642 | 0.2829 | 0.2661 |
| rs7688167 | 9893506 | A | 0.4388 | 0.300747 | -0.1093 | 0.1056 |
| rs13135351 | 10033525 | A | 0.1081 | 0.301657 | -0.0737 | 0.0714 |
| rs3822250 | 10036630 | T | 0.1081 | 0.301657 | -0.0737 | 0.0714 |
| rs112215724 | 9927690 | T | 0.0062 | 0.308351 | -0.3523 | 0.3459 |
| rs113995811 | 9996604 | T | 0.0062 | 0.308351 | -0.3523 | 0.3459 |
| rs73096620 | 9932341 | A | 0.0062 | 0.308351 | -0.3523 | 0.3459 |
| rs73096634 | 9935584 | A | 0.0062 | 0.308351 | -0.3523 | 0.3459 |
| rs76800309 | 9991723 | T | 0.0062 | 0.308351 | -0.3523 | 0.3459 |
| rs77275218 | 9986616 | T | 0.0062 | 0.308351 | -0.3523 | 0.3459 |
| rs77935410 | 9923008 | G | 0.0062 | 0.308351 | -0.3523 | 0.3459 |
| rs79704809 | 9938279 | A | 0.0062 | 0.308351 | -0.3523 | 0.3459 |
| rs114663735 | 9992998 | C | 0.0062 | 0.308455 | -0.3524 | 0.3460 |
| rs77459722 | 9947200 | A | 0.1251 | 0.32097 | -0.0662 | 0.0667 |
| rs28489733 | 9892061 | A | 0.0796 | 0.3225 | 0.0820 | 0.0829 |
| rs58769709 | 9891240 | A | 0.0796 | 0.3225 | 0.0820 | 0.0829 |
| rs59815647 | 9891009 | A | 0.0796 | 0.322982 | 0.0820 | 0.0830 |
| rs116108158 | 10026574 | C | 0.0041 | 0.324681 | 0.3874 | 0.3933 |
| rs141899952 | 10042458 | G | 0.0014 | 0.328479 | 0.5812 | 0.5948 |
| rs71603975 | 9937741 | T | 0.1214 | 0.331455 | -0.0655 | 0.0675 |
| rs112404957 | 9922187 | A | 0.0035 | 0.338293 | 0.2993 | 0.3126 |
| rs143903193 | 10013481 | A | 0.0028 | 0.356109 | -0.4817 | 0.5220 |
| rs144402103 | 10029470 | G | 0.0028 | 0.356109 | -0.4817 | 0.5220 |
| rs41268375 | 10036359 | C | 0.0028 | 0.356109 | -0.4817 | 0.5220 |
| rs77862592 | 9935234 | T | 0.1711 | 0.370858 | 0.0539 | 0.0603 |
| rs56036138 | 9894954 | A | 0.0749 | 0.371339 | 0.0753 | 0.0843 |
| rs16891285 | 9954630 | C | 0.1239 | 0.372668 | -0.0600 | 0.0673 |
| rs4697909 | 9896760 | A | 0.1180 | 0.374853 | 0.0631 | 0.0711 |
| rs368486099 | 9828003 | T | 0.0090 | 0.376303 | -0.2391 | 0.2703 |
| rs78801470 | 9947090 | A | 0.1245 | 0.381059 | -0.0587 | 0.0670 |
| rs4345181 | 10026864 | T | 0.1042 | 0.386397 | -0.0628 | 0.0725 |
| rs141224249 | 9990429 | G | 0.0069 | 0.389762 | -0.2777 | 0.3229 |
| rs114756544 | 9932882 | T | 0.0117 | 0.394851 | 0.1953 | 0.2295 |
| rs115662655 | 9948527 | C | 0.0014 | 0.39566 | 0.6041 | 0.7112 |
| rs7685958 | 10042850 | G | 0.2227 | 0.428706 | 0.0528 | 0.0668 |
| rs58871485 | 9954859 | G | 0.0021 | 0.449135 | -0.4201 | 0.5551 |
| rs938557 | 9827556 | T | 0.0639 | 0.45204 | 0.0721 | 0.0958 |
| rs10516197 | 10042100 | T | 0.2648 | 0.45571 | 0.0395 | 0.0530 |
| rs16892449 | 10038591 | C | 0.2654 | 0.459536 | 0.0391 | 0.0529 |
| rs114047413 | 9891525 | A | 0.0014 | 0.460052 | 0.4105 | 0.5557 |
| rs115502819 | 9935277 | C | 0.0028 | 0.465886 | -0.3845 | 0.5273 |
| rs57477583 | 9939648 | T | 0.0035 | 0.47044 | 0.2344 | 0.3248 |
| rs35249656 | 10038542 | A | 0.2657 | 0.477509 | 0.0376 | 0.0529 |
| rs116388124 | 9890133 | G | 0.0028 | 0.491877 | 0.3539 | 0.5150 |
| rs3733591 | 9922130 | T | 0.1968 | 0.535647 | 0.0412 | 0.0665 |
| rs138339252 | 9891440 | C | 0.0048 | 0.537392 | 0.2199 | 0.3565 |
| rs7684245 | 9895180 | T | 0.0069 | 0.543798 | -0.1814 | 0.2988 |
| rs115588006 | 10030411 | G | 0.0062 | 0.546017 | 0.1983 | 0.3285 |
| rs10939614 | 9926613 | C | 0.4885 | 0.556744 | -0.0980 | 0.1668 |
| rs147002367 | 9993348 | T | 0.0041 | 0.560725 | 0.2295 | 0.3944 |
| rs35922993 | 9925925 | T | 0.1241 | 0.562069 | -0.0388 | 0.0668 |
| rs1519092 | 9826801 | T | 0.0645 | 0.563189 | 0.0552 | 0.0955 |
| rs74794351 | 10027664 | G | 0.0014 | 0.572958 | 0.4096 | 0.7267 |
| rs73096647 | 9940994 | A | 0.0035 | 0.587747 | -0.2497 | 0.4605 |
| rs138206300 | 9993426 | A | 0.0007 | 0.599609 | -0.5545 | 1.0562 |
| rs141090791 | 10038434 | A | 0.0035 | 0.610785 | -0.2177 | 0.4277 |
| rs187024415 | 9926934 | A | 0.0022 | 0.621032 | -0.2247 | 0.4544 |
| rs189741361 | 9898916 | C | 0.0035 | 0.632199 | -0.1770 | 0.3699 |
| rs147139740 | 9953364 | G | 0.0014 | 0.641197 | -0.3420 | 0.7338 |
| rs78755014 | 9896571 | C | 0.0007 | 0.682363 | -0.4321 | 1.0560 |
| rs149022308 | 10033810 | C | 0.0021 | 0.712869 | -0.1873 | 0.5090 |
| rs60956448 | 9927498 | T | 0.0021 | 0.758065 | -0.1693 | 0.5497 |
| rs137912718 | 9937349 | A | 0.0021 | 0.75883 | -0.1329 | 0.4330 |
| rs115251876 | 9935012 | T | 0.0014 | 0.787035 | -0.1761 | 0.6518 |
| rs59142033 | 9890201 | A | 0.0062 | 0.79984 | -0.0776 | 0.3061 |
| rs12505366 | 9897520 | A | 0.1517 | 0.810926 | -0.0170 | 0.0712 |
| rs62294288 | 9931173 | T | 0.0049 | 0.813193 | -0.0784 | 0.3319 |
| rs60959435 | 10020896 | C | 0.0035 | 0.813416 | 0.0934 | 0.3958 |
| rs113006138 | 9891941 | C | 0.0014 | 0.816123 | 0.1672 | 0.7191 |
| rs16891923 | 9996661 | A | 0.0941 | 0.854056 | -0.0141 | 0.0766 |
| rs16891926 | 9996852 | T | 0.0941 | 0.854056 | -0.0141 | 0.0766 |
| rs183034270 | 9935793 | C | 0.0007 | 0.857524 | 0.1044 | 0.5817 |
| rs139291984 | 9892172 | A | 0.0014 | 0.869909 | -0.1091 | 0.6660 |
| rs78698809 | 10013759 | A | 0.0014 | 0.869909 | -0.1091 | 0.6660 |
| rs113692117 | 9931902 | G | 0.0028 | 0.870251 | 0.0697 | 0.4264 |
| rs73100648 | 9995473 | T | 0.0028 | 0.870251 | 0.0697 | 0.4264 |
| rs10012779 | 10038112 | T | 0.0256 | 0.894399 | -0.0203 | 0.1528 |
| rs10005326 | 9938591 | A | 0.0042 | 0.925933 | -0.0343 | 0.3690 |
| rs79551626 | 9932253 | C | 0.0028 | 0.929498 | 0.0450 | 0.5085 |
| rs73225891 | 9922170 | G | 0.0056 | 0.940103 | 0.0255 | 0.3392 |
| rs79679425 | 9953853 | C | 0.0014 | 0.941464 | -0.0546 | 0.7439 |
| rs143022050 | 10033811 | G | 0.0021 | 0.944001 | 0.0429 | 0.6107 |
| rs145154883 | 9896609 | A | 0.0021 | 0.944001 | 0.0429 | 0.6107 |
| rs10939654 | 10013344 | G | 0.0256 | 0.946635 | 0.0101 | 0.1509 |
| rs75245044 | 10042686 | A | 0.0035 | 0.956965 | -0.0164 | 0.3041 |
| rs184752109 | 9947255 | G | 0.0007 | 0.965618 | -0.0455 | 1.0563 |
| rs114495476 | 9893262 | G | 0.0014 | 0.997477 | -0.0021 | 0.6680 |
| rs116652724 | 9938010 | A | 0.0014 | 0.997477 | -0.0021 | 0.6680 |
| rs141955300 | 9938273 | G | 0.0014 | 0.997477 | -0.0021 | 0.6680 |

SNP: single nucleotide polymorphism; Chr pos: chromosome position in base pairs; MAF: minor allele frequency; P-value: P-values from measured genotype analysis; β: beta coefficient of the SNP; SE: standard error; Effect Size: Proportion of the residual phenotypic variance that is explained by the minor allele of the SNP

**Supplementary Table 3. Association of *SLC2A9* sequence variants and SU in each of the three centers of SHFS. Associations that are significant at p < 2 x 10^-4^ in at least one center are shown below**

|  | **Oklahoma** | | | **Dalotas** | | | **Arizona** | | |
| --- | --- | --- | --- | --- | --- | --- | --- | --- | --- |
| **SNP** | **P value** | **β** | **SE** | **P value** | **β** | **SE** | **P value** | **β** | **SE** |
| rs11723439 | 7.08E-15 | -0.36 | 0.05 | 8.89E-11 | -0.33 | 0.05 | - | - | - |
| rs4481233 | 1.21E-14 | -0.36 | 0.05 | 1.82E-11 | -0.34 | 0.05 | - | - | - |
| rs4428284 | 7.26E-14 | -0.36 | 0.05 | 1.41E-09 | -0.36 | 0.06 | - | - | - |
| rs13111638 | 1.05E-13 | -0.36 | 0.05 | 1.53E-09 | -0.35 | 0.06 | - | - | - |
| rs28592748 | 1.13E-13 | -0.35 | 0.05 | 2.64E-10 | -0.36 | 0.06 | - | - | - |
| rs7678287 | 1.38E-13 | -0.35 | 0.05 | 3.55E-10 | -0.36 | 0.06 | - | - | - |
| rs7669607 | 2.55E-13 | -0.35 | 0.05 | 3.98E-10 | -0.36 | 0.06 | - | - | - |
| rs7683856 | 3.52E-13 | -0.35 | 0.05 | 3.15E-08 | -0.33 | 0.06 | - | - | - |
| rs4385059 | 9.22E-13 | -0.34 | 0.05 | 4.70E-09 | -0.34 | 0.06 | - | - | - |
| rs4637402 | 1.14E-12 | -0.33 | 0.05 | 2.91E-08 | -0.32 | 0.06 | - | - | - |
| rs12509955 | 2.24E-12 | -0.33 | 0.05 | 9.75E-09 | -0.33 | 0.06 | - | - | - |
| rs9291642 | 3.88E-12 | -0.34 | 0.05 | 2.79E-08 | -0.34 | 0.06 | - | - | - |
| rs3775946 | 4.41E-12 | -0.30 | 0.04 | 2.75E-10 | -0.33 | 0.05 | - | - | - |
| rs4144 | 4.99E-12 | -0.33 | 0.05 | 2.91E-08 | -0.32 | 0.06 | - | - | - |
| rs4447862 | 5.95E-12 | 0.31 | 0.05 | 3.86E-08 | 0.25 | 0.05 | 0.000037 | 0.43 | 0.10 |
| rs6449137 | 6.72E-12 | 0.31 | 0.05 | 3.54E-08 | 0.25 | 0.05 | 0.000036 | 0.43 | 0.10 |
| rs10939650 | 7.20E-12 | -0.30 | 0.04 | 3.77E-10 | -0.32 | 0.05 | - | - | - |
| rs938558 | 1.07E-11 | 0.31 | 0.05 | 4.43E-08 | 0.25 | 0.05 | 0.000036 | 0.43 | 0.10 |
| rs3775948 | 1.09E-11 | -0.30 | 0.04 | 5.80E-10 | -0.32 | 0.05 | - | - | - |
| rs3733588 | 1.22E-11 | -0.30 | 0.04 | 3.77E-10 | -0.32 | 0.05 | - | - | - |
| rs4591605 | 1.37E-11 | -0.35 | 0.05 | 1.39E-09 | -0.37 | 0.06 | - | - | - |
| rs6827401 | 1.50E-11 | -0.33 | 0.05 | 1.21E-07 | -0.32 | 0.06 | - | - | - |
| rs938554 | 1.56E-11 | -0.29 | 0.04 | 1.72E-08 | -0.26 | 0.05 | - | - | - |
| rs874432 | 1.56E-11 | -0.29 | 0.04 | 3.55E-08 | -0.26 | 0.05 | - | - | - |
| rs4697701 | 1.78E-11 | 0.31 | 0.05 | 6.98E-08 | 0.24 | 0.05 | 0.000048 | 0.43 | 0.11 |
| rs938555 | 1.89E-11 | -0.30 | 0.04 | 1.98E-08 | -0.26 | 0.05 | - | - | - |
| rs3796841 | 2.02E-11 | -0.33 | 0.05 | 1.87E-08 | -0.33 | 0.06 | - | - | - |
| rs1014290 | 2.13E-11 | -0.30 | 0.04 | 1.98E-10 | -0.33 | 0.05 | - | - | - |
| rs13115469 | 2.20E-11 | -0.29 | 0.04 | 2.94E-08 | -0.26 | 0.05 | - | - | - |
| rs734553 | 2.47E-11 | -0.29 | 0.04 | 5.30E-09 | -0.27 | 0.05 | - | - | - |
| rs4529048 | 2.58E-11 | -0.30 | 0.04 | 1.17E-10 | -0.33 | 0.05 | - | - | - |
| rs7660895 | 4.08E-11 | 0.29 | 0.04 | 1.29E-07 | 0.24 | 0.05 | 0.000015 | 0.45 | 0.10 |
| rs6849717 | 4.81E-11 | 0.31 | 0.05 | 1.72E-06 | 0.22 | 0.05 | 0.000062 | 0.43 | 0.11 |
| rs34709913 | 6.56E-11 | 0.29 | 0.04 | 4.62E-07 | -0.25 | 0.05 | - | - | - |
| rs3733585 | 6.79E-11 | 0.29 | 0.04 | 4.12E-07 | -0.25 | 0.05 | - | - | - |
| rs998675 | 7.06E-11 | 0.31 | 0.05 | 1.59E-06 | 0.22 | 0.05 | 0.000062 | 0.43 | 0.11 |
| rs7376960 | 7.07E-11 | -0.29 | 0.04 | 2.59E-07 | -0.24 | 0.05 | - | - | - |
| rs3733584 | 7.30E-11 | 0.29 | 0.04 | 3.24E-07 | -0.25 | 0.05 | - | - | - |
| rs4447863 | 7.68E-11 | 0.31 | 0.05 | 2.03E-06 | 0.22 | 0.05 | - | - | - |
| rs11942223 | 8.33E-11 | -0.28 | 0.04 | 1.14E-07 | -0.24 | 0.05 | - | - | - |
| rs7442295 | 8.34E-11 | -0.28 | 0.04 | 4.59E-08 | -0.25 | 0.05 | - | - | - |
| rs55959894 | 8.66E-11 | 0.29 | 0.04 | 4.62E-07 | -0.25 | 0.05 | - | - | - |
| rs998676 | 8.68E-11 | 0.32 | 0.05 | 6.02E-06 | 0.21 | 0.05 | 0.000262 | 0.40 | 0.11 |
| rs7663044 | 9.79E-11 | -0.32 | 0.05 | 9.83E-08 | -0.33 | 0.06 | - | - | - |
| rs7696092 | 1.03E-10 | -0.31 | 0.05 | 1.06E-07 | -0.31 | 0.06 | - | - | - |
| rs13139055 | 1.05E-10 | -0.29 | 0.05 | 3.41E-06 | -0.24 | 0.05 | - | - | - |
| rs2240722 | 1.08E-10 | 0.29 | 0.04 | 3.94E-07 | -0.25 | 0.05 | - | - | - |
| rs7663032 | 1.11E-10 | -0.29 | 0.04 | 1.44E-09 | -0.31 | 0.05 | - | - | - |
| rs6449173 | 1.30E-10 | -0.28 | 0.04 | 4.59E-08 | -0.25 | 0.05 | - | - | - |
| rs9998811 | 1.35E-10 | -0.28 | 0.04 | 3.98E-08 | -0.25 | 0.05 | - | - | - |
| rs7678012 | 1.47E-10 | 0.28 | 0.04 | 2.97E-07 | -0.25 | 0.05 | - | - | - |
| rs4697700 | 1.50E-10 | -0.28 | 0.04 | 3.58E-07 | -0.23 | 0.05 | - | - | - |
| rs4280729 | 1.60E-10 | 0.28 | 0.04 | 3.61E-07 | -0.25 | 0.05 | - | - | - |
| rs12507606 | 1.72E-10 | -0.27 | 0.04 | 2.03E-07 | -0.24 | 0.05 | - | - | - |
| rs7670751 | 1.75E-10 | -0.28 | 0.04 | 7.31E-08 | -0.24 | 0.05 | - | - | - |
| rs10010582 | 1.80E-10 | -0.27 | 0.04 | 1.33E-07 | -0.24 | 0.05 | - | - | - |
| rs13106991 | 1.84E-10 | -0.27 | 0.04 | 1.37E-07 | -0.24 | 0.05 | - | - | - |
| rs10017945 | 1.93E-10 | -0.28 | 0.04 | 7.31E-08 | -0.24 | 0.05 | - | - | - |
| rs17187075 | 1.98E-10 | 0.28 | 0.04 | 3.93E-07 | -0.25 | 0.05 | - | - | - |
| rs34501273 | 2.00E-10 | 0.28 | 0.04 | 4.99E-07 | -0.25 | 0.05 | - | - | - |
| rs3796842 | 2.38E-10 | 0.28 | 0.04 | 5.22E-07 | -0.25 | 0.05 | - | - | - |
| rs2240721 | 2.47E-10 | 0.28 | 0.04 | 4.06E-07 | -0.25 | 0.05 | - | - | - |
| rs7679916 | 2.56E-10 | 0.32 | 0.05 | 3.96E-06 | 0.22 | 0.05 | - | - | - |
| rs12499857 | 2.76E-10 | -0.29 | 0.05 | 1.08E-06 | -0.25 | 0.05 | - | - | - |
| rs3756236 | 2.77E-10 | 0.28 | 0.04 | 4.30E-07 | -0.25 | 0.05 | - | - | - |
| rs4697699 | 2.81E-10 | -0.27 | 0.04 | 2.27E-07 | -0.24 | 0.05 | - | - | - |
| rs6855911 | 2.90E-10 | -0.27 | 0.04 | 7.71E-08 | -0.24 | 0.05 | - | - | - |
| rs6449139 | 2.95E-10 | -0.27 | 0.04 | 7.71E-08 | -0.24 | 0.05 | - | - | - |
| rs3756237 | 3.46E-10 | 0.28 | 0.04 | 5.16E-07 | -0.25 | 0.05 | - | - | - |
| rs10516194 | 4.24E-10 | -0.32 | 0.05 | 1.33E-07 | -0.30 | 0.06 | - | - | - |
| rs13137069 | 4.45E-10 | -0.27 | 0.04 | 2.27E-07 | -0.24 | 0.05 | - | - | - |
| rs11722229 | 4.84E-10 | -0.27 | 0.04 | 7.59E-08 | -0.25 | 0.05 | - | - | - |
| rs1071988 | 4.86E-10 | -0.27 | 0.04 | 6.83E-08 | -0.25 | 0.05 | - | - | - |
| rs751092 | 6.00E-10 | -0.27 | 0.04 | 1.32E-08 | -0.31 | 0.05 | - | - | - |
| rs13137074 | 6.23E-10 | 0.29 | 0.05 | 0.000014 | 0.20 | 0.05 | - | - | - |
| rs6845554 | 6.32E-10 | -0.28 | 0.05 | 2.57E-06 | -0.25 | 0.05 | - | - | - |
| rs6856127 | 6.50E-10 | 0.29 | 0.05 | 5.42E-06 | 0.24 | 0.05 | - | - | - |
| rs10023068 | 6.99E-10 | -0.27 | 0.04 | 4.39E-09 | -0.31 | 0.05 | - | - | - |
| rs10016075 | 7.06E-10 | -0.27 | 0.04 | 6.50E-09 | -0.31 | 0.05 | - | - | - |
| rs13101785 | 7.16E-10 | 0.29 | 0.05 | 0.000014 | 0.20 | 0.05 | - | - | - |
| rs16868246 | 7.29E-10 | -0.27 | 0.04 | 6.13E-08 | -0.25 | 0.05 | - | - | - |
| rs56113653 | 8.84E-10 | -0.28 | 0.05 | - | - | - | - | - | - |
| rs13125646 | 8.94E-10 | -0.27 | 0.04 | 4.33E-08 | -0.25 | 0.05 | - | - | - |
| rs7439210 | 9.65E-10 | -0.27 | 0.04 | 5.87E-08 | -0.25 | 0.05 | - | - | - |
| rs9291640 | 1.01E-09 | -0.27 | 0.04 | 1.52E-08 | -0.30 | 0.05 | - | - | - |
| rs13145758 | 1.13E-09 | -0.27 | 0.04 | 6.92E-08 | -0.25 | 0.05 | - | - | - |
| rs7682751 | 1.23E-09 | -0.27 | 0.04 | 1.08E-07 | -0.24 | 0.05 | - | - | - |
| rs6838021 | 1.31E-09 | -0.26 | 0.04 | 7.85E-07 | -0.23 | 0.05 | 0.00037 | 0.35 | 0.10 |
| rs7435196 | 1.38E-09 | 0.26 | 0.04 | 1.73E-06 | 0.22 | 0.05 | - | - | - |
| rs6823324 | 1.43E-09 | -0.27 | 0.04 | 5.11E-08 | -0.25 | 0.05 | 0.000434 | 0.33 | 0.10 |
| rs28837683 | 1.69E-09 | 0.26 | 0.04 | 5.01E-06 | 0.21 | 0.05 | - | - | - |
| rs10022499 | 1.71E-09 | -0.27 | 0.04 | 4.01E-08 | -0.30 | 0.05 | - | - | - |
| rs11724510 | 1.74E-09 | 0.26 | 0.04 | 4.77E-06 | 0.21 | 0.05 | - | - | - |
| rs6449154 | 1.75E-09 | 0.26 | 0.04 | 2.87E-06 | 0.22 | 0.05 | - | - | - |
| rs9994266 | 1.78E-09 | 0.26 | 0.04 | 0.000011 | 0.20 | 0.05 | - | - | - |
| rs7672947 | 1.79E-09 | 0.26 | 0.04 | 4.48E-06 | 0.21 | 0.05 | - | - | - |
| rs6449176 | 1.85E-09 | 0.26 | 0.04 | 1.84E-06 | 0.22 | 0.05 | - | - | - |
| rs7683283 | 1.85E-09 | 0.26 | 0.04 | 1.88E-06 | 0.22 | 0.05 | - | - | - |
| rs4311316 | 1.94E-09 | 0.26 | 0.04 | 4.39E-06 | 0.21 | 0.05 | - | - | - |
| rs6449155 | 1.94E-09 | 0.26 | 0.04 | 4.48E-06 | 0.21 | 0.05 | - | - | - |
| rs6834893 | 1.94E-09 | 0.26 | 0.04 | 4.39E-06 | 0.21 | 0.05 | - | - | - |
| rs4459990 | 1.95E-09 | 0.26 | 0.04 | 0.000011 | 0.20 | 0.05 | - | - | - |
| rs6843873 | 1.96E-09 | 0.26 | 0.04 | 4.39E-06 | 0.21 | 0.05 | - | - | - |
| rs7676733 | 1.99E-09 | 0.26 | 0.04 | 1.84E-06 | 0.22 | 0.05 | - | - | - |
| rs7677710 | 2.01E-09 | 0.26 | 0.04 | 1.88E-06 | 0.22 | 0.05 | - | - | - |
| rs7658170 | 2.10E-09 | 0.26 | 0.04 | 1.88E-06 | 0.22 | 0.05 | - | - | - |
| rs10939605 | 2.12E-09 | -0.28 | 0.05 | - | - | - | - | - | - |
| rs56038393 | 2.12E-09 | -0.28 | 0.05 | - | - | - | - | - | - |
| rs6850684 | 2.26E-09 | 0.26 | 0.04 | 4.24E-06 | 0.21 | 0.05 | - | - | - |
| rs4235347 | 2.27E-09 | 0.26 | 0.04 | 0.000011 | 0.20 | 0.05 | - | - | - |
| rs6449202 | 2.29E-09 | 0.26 | 0.04 | 2.10E-06 | 0.22 | 0.05 | - | - | - |
| rs7434391 | 2.29E-09 | 0.26 | 0.04 | 1.88E-06 | 0.22 | 0.05 | - | - | - |
| rs4697914 | 2.29E-09 | 0.26 | 0.04 | 0.000013 | 0.20 | 0.05 | - | - | - |
| rs6826764 | 2.35E-09 | -0.26 | 0.04 | 4.54E-08 | -0.29 | 0.05 | - | - | - |
| rs6850143 | 2.37E-09 | 0.26 | 0.04 | 3.69E-06 | 0.21 | 0.05 | - | - | - |
| rs4697697 | 2.45E-09 | 0.26 | 0.04 | 0.00009 | 0.18 | 0.05 | - | - | - |
| rs10018204 | 2.48E-09 | 0.26 | 0.04 | 4.33E-06 | 0.21 | 0.05 | - | - | - |
| rs7699609 | 2.57E-09 | 0.26 | 0.04 | 5.74E-06 | 0.21 | 0.05 | - | - | - |
| rs6449159 | 2.67E-09 | 0.25 | 0.04 | 4.80E-06 | 0.21 | 0.05 | - | - | - |
| rs4312757 | 2.88E-09 | 0.26 | 0.04 | 5.74E-06 | 0.21 | 0.05 | - | - | - |
| rs6449178 | 3.18E-09 | 0.25 | 0.04 | 1.84E-06 | 0.22 | 0.05 | - | - | - |
| rs17245723 | 3.25E-09 | 0.25 | 0.04 | 3.47E-06 | 0.21 | 0.05 | - | - | - |
| rs4292332 | 3.29E-09 | 0.25 | 0.04 | 0.000011 | 0.20 | 0.05 | - | - | - |
| rs4473653 | 3.36E-09 | 0.25 | 0.04 | 1.88E-06 | 0.22 | 0.05 | - | - | - |
| rs62294331 | 3.37E-09 | 0.25 | 0.04 | 4.69E-06 | 0.21 | 0.05 | - | - | - |
| rs6449201 | 3.44E-09 | 0.25 | 0.04 | 1.84E-06 | 0.22 | 0.05 | - | - | - |
| rs76574510 | 4.11E-09 | 0.25 | 0.04 | 0.000013 | 0.20 | 0.05 | - | - | - |
| rs12498150 | 4.76E-09 | 0.25 | 0.04 | 6.70E-06 | 0.21 | 0.05 | - | - | - |
| rs13328050 | 4.92E-09 | 0.25 | 0.04 | 6.36E-06 | 0.21 | 0.05 | - | - | - |
| rs7376948 | 5.03E-09 | 0.25 | 0.04 | 0.000011 | 0.20 | 0.05 | - | - | - |
| rs6449172 | 5.29E-09 | 0.25 | 0.04 | 1.82E-06 | 0.22 | 0.05 | - | - | - |
| rs11381848 | 5.53E-09 | 0.25 | 0.04 | 2.44E-06 | 0.22 | 0.05 | - | - | - |
| rs60045583 | 6.22E-09 | 0.25 | 0.04 | 1.54E-06 | 0.22 | 0.05 | - | - | - |
| rs1079128 | 6.44E-09 | 0.25 | 0.04 | 6.49E-06 | 0.21 | 0.05 | - | - | - |
| rs6853437 | 7.11E-09 | -0.26 | 0.05 | 1.46E-08 | -0.30 | 0.05 | - | - | - |
| rs4621431 | 7.78E-09 | 0.25 | 0.04 | 9.08E-06 | 0.20 | 0.05 | - | - | - |
| rs4580649 | 8.27E-09 | 0.25 | 0.04 | 6.53E-06 | 0.21 | 0.05 | - | - | - |
| rs4697693 | 9.52E-09 | -0.27 | 0.05 | - | - | - | - | - | - |
| rs7694997 | 1.12E-08 | 0.25 | 0.04 | 9.30E-06 | 0.20 | 0.05 | - | - | - |
| rs4447861 | 1.35E-08 | 0.25 | 0.04 | 0.000035 | 0.19 | 0.05 | - | - | - |
| rs7378305 | 1.50E-08 | 0.25 | 0.04 | 0.000012 | 0.21 | 0.05 | - | - | - |
| rs13103497 | 3.22E-08 | 0.24 | 0.04 | 2.54E-06 | -0.22 | 0.05 | - | - | - |
| rs13145554 | 5.50E-08 | 0.23 | 0.04 | 3.47E-06 | -0.21 | 0.05 | - | - | - |
| rs35954357 | 6.84E-08 | 0.23 | 0.04 | 2.63E-06 | -0.22 | 0.05 | - | - | - |
| rs6449179 | 6.86E-08 | -0.24 | 0.04 | 0.000019 | -0.20 | 0.05 | - | - | - |
| rs6839490 | 8.90E-08 | -0.24 | 0.04 | 6.87E-06 | 0.21 | 0.05 | - | - | - |
| rs7349721 | 1.01E-07 | 0.23 | 0.04 | 0.000015 | -0.20 | 0.05 | - | - | - |
| rs4697893 | 1.53E-07 | -0.27 | 0.05 | - | - | - | - | - | - |
| rs6823877 | 2.50E-07 | 0.27 | 0.05 | 9.43E-07 | 0.24 | 0.05 | 0.000225 | 0.44 | 0.12 |
| rs4621429 | 3.96E-07 | -0.22 | 0.04 | - | - | - | - | - | - |
| rs78291681 | 1.00E-06 | -0.22 | 0.05 | 0.000075 | 0.19 | 0.05 | - | - | - |
| rs10939614 | 1.09E-06 | 0.28 | 0.06 | 0.000142 | 0.20 | 0.05 | - | - | - |
| rs12644047 | 2.43E-06 | -0.20 | 0.04 | - | - | - | - | - | - |
| rs1107710 | 2.46E-06 | -0.20 | 0.04 | 0.000295 | 0.17 | 0.05 | - | - | - |
| rs6449144 | 6.63E-06 | 0.26 | 0.06 | 0.000318 | 0.20 | 0.05 | - | - | - |
| rs7669444 | 6.79E-06 | 0.20 | 0.04 | - | - | - | - | - | - |
| rs7688167 | 7.28E-06 | 0.20 | 0.04 | - | - | - | - | - | - |
| rs10939663 | 0.000027 | 0.27 | 0.06 | - | - | - | - | - | - |
| rs938563 | 0.000031 | 0.18 | 0.04 | - | - | - | - | - | - |
| rs4697692 | 0.000046 | -0.17 | 0.04 | - | - | - | - | - | - |
| rs7698858 | 0.000091 | -0.17 | 0.04 | 0.000251 | -0.16 | 0.04 | - | - | - |
